# Tailor-made porosities of fluorene-based porous organic frameworks for the pre-designable fabrication of palladium nanoparticles with size, location and distribution control†
†Electronic supplementary information (ESI) available: Detailed experimental section for the materials, and the general synthetic procedures for the building blocks and the polymer frameworks; TGA, FT-IR spectra, solid state NMR spectra, SEM images, additional gas adsorption data, and theoretical calculations. See DOI: 10.1039/c5sc04351d


**DOI:** 10.1039/c5sc04351d

**Published:** 2015-12-10

**Authors:** Hong Zhong, Caiping Liu, Yangxin Wang, Ruihu Wang, Maochun Hong

**Affiliations:** a State Key Laboratory of Structural Chemistry , Fujian Institute of Research on the Structure of Matter , Chinese Academy of Sciences , Fuzhou , Fujian 350002 , China . Email: ruihu@fjirsm.ac.cn

## Abstract

Porous organic frameworks (POFs) are a promising new class of support for metal nanoparticles (NPs), with the size, location and distribution of metal NPs are closely related to the porous nature of the POFs. In this contribution, three fluorene-based POFs containing coordination-inert hydrogen, propyl and benzyl substituents at the 9-position of the fluorene units (POF-1, POF-2 and POF-3) were synthesized through a simple click reaction. The substituents exerted important influences on the surface area, pore volume and pore size of the POFs. Palladium NPs with a pre-designable size, location and distribution were synthesized through a substituent-controlled strategy. When POF-1 was employed as a support, ultrafine palladium NPs in the interior pores were generated, while the introduction of propyl at the 9-position of fluorene in POF-2 gave rise to dual-distributed palladium NPs in the interior pores and on the external surface. The use of the bulkier benzyl substituent resulted in the formation of palladium NPs on the external surface of POF-3. The hydrogenation of olefins has demonstrated that palladium NPs on the external surface possessed higher catalytic activity, while palladium NPs in the interior pores exhibited higher stability and recyclability. In addition, after Pd/POF-1, Pd/POF-2 and Pd/POF-3 were stored in air over half a year, palladium NPs in the interior pores showed a negligible change in comparison with fresh samples, while an obvious agglomeration was observed for palladium NPs on the external surface.

## Abstract 2

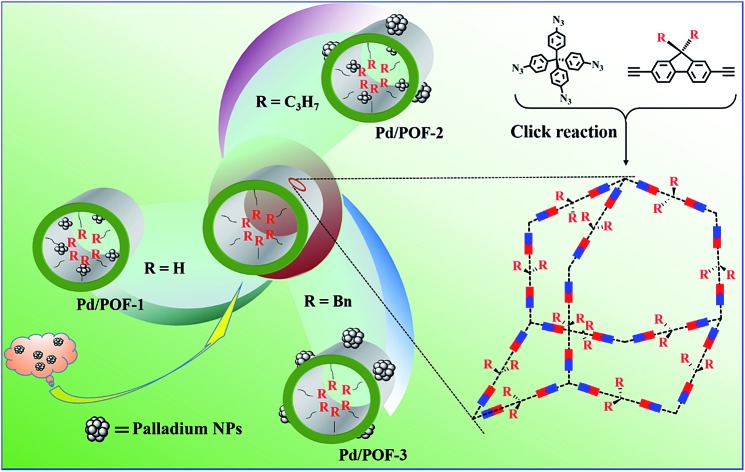
Supported palladium nanoparticles with pre-designable size, location and distribution were presented through tailor-made porosities of fluorene-based porous organic frameworks.

## Introduction

Porous organic frameworks (POFs) have attracted considerable interest for gas storage, drug delivery, sensing and heterogeneous catalysis owing to their large surface areas, flexible synthetic strategies and high stabilities.^1^–^5^ They can serve as promising supports for metal nanoparticles (NPs) based on their powerful confinement effects. Metal NPs are usually encapsulated in the interior pores or dispersed on the external surface, or dually distributed on the external surface and in the interior pores of the POFs.^6^–^10^ A variety of approaches, including the introduction of coordination groups^11^–^14^ and the judicial selection of the reductive method of metal precursors,^15^ have been developed, but metal NPs with pre-designable sizes, locations and distributions are still not predictably fabricated. The controllable synthesis of metal NPs is regarded as one of the priorities for the development of highly efficient catalytic systems and is inevitably tied to the search for new supports with unique structures and properties.^16^–^18^ Therefore, it is a great challenge to establish a general and facile POF platform which allows the flexible adjustment of the size, location and distribution of the metal NPs immobilized by the POF.

As is well known, the properties of metal NPs are closely related to porous nature of POFs, one of the feasible solutions for tailor-made porosities of POFs is to use a strategy of post-synthetic modification.^19^–^28^ Recently, Zhu *et al.* adjusted the pores of cationic POFs by changing the size of the exchanged anions for the selective adsorption of gas molecules.^24^ Zhou *et al.* modified POFs using alkyl amino and sulfate groups to increase the gas adsorption ability.^25^,^26^ Jiang *et al.* accomplished the functionalization of surface pores by facile click reactions between azides and alkynes.^27^,^28^ However, their applications have mainly focused on gas sorption and separation, the exploration of supports for metal NPs for heterogeneous catalysis is seldom reported. In addition, the random incorporation of the introduced groups is unfavorable for the precise tuning of the size, location and distribution of the metal NPs.^29^,^30^ In contrast, the modification of the building units of the POFs at the molecular level is more reliable for modulating the pore size and surface properties, moreover, the inherent electronic and steric properties of the introduced groups may deliberately balance the interactions between the POFs and metal NPs, and finally endow the metal NPs with unique physicochemical and catalytic properties.^31^–^33^ The selection of the modifiable building units is crucial for the construction of POFs with optimal porous natures. Although the tuning of the surface area, pore volume and pore size of POFs has been achieved through varying the length of the organic linkages and selecting suitable synthetic methods,^34^–^39^ it is still imperative to develop a general and systematic method for tailor-made porosities to establish a well-defined relationship between the porosities of POFs and the supported palladium NPs.

As a kind of distinctive electron-rich aromatic compound, fluorene possesses attractive characteristics for structural modification, it is ready to polymerize at positions 2 and 7 through various chemical reactions, while functionalization at the 9-position may modify the properties of the resulting polymers for a specific purpose.^40^–^45^ Many fluorene-based supramolecular architectures and optoelectronic materials have been presented,^41^,^44^ but the application of fluorene-based POFs as stabilizers of metal NPs has not been reported hitherto. Herein, we chose 9-position-substituted fluorenes as monomers and present three fluorene-based POFs containing coordination-inert hydrogen, propyl and benzyl substituents (POF-1, POF-2 and POF-3). The synthesis of size, location and distribution controlled palladium NPs in the POFs was achieved for the first time by the tailoring porous nature of the POFs using a substituent-control strategy.

## Results and discussion

**Scheme 1:**
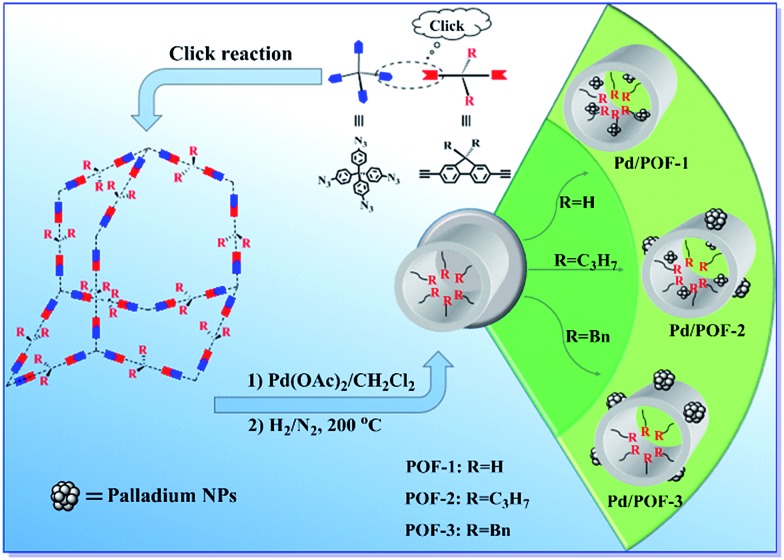
Schematic synthesis of POF-1, POF-2, POF-3, Pd/POF-1, Pd/POF-2 and Pd/POF-3.

POF-1, POF-2 and POF-3 were readily prepared by click reactions between tetrakis(4-azidophenyl)methane and 9-position-substituted 2,7-diethynylfluorene (R = H, propyl and benzyl) under standard click conditions (Scheme 1). After the reaction, the precipitates were collected by filtration and washed successively with aqueous EDTA–2Na solution, ethanol and CH_2_Cl_2_ to remove any possible residues. The resultant deep yellow powders were further treated by Soxhlet extraction in CH_2_Cl_2_ and dried *in vacuo* at 80 °C for 12 h.

POF-1, POF-2 and POF-3 are stable in water and in an air atmosphere, their structures and compositions were defined by FTIR, solid-state ^13^C NMR and elemental analysis. In the FTIR spectra (Fig. S1†), the characteristic peaks of the azido at 2121 cm^–1^ and terminal alkynyl around 3280 and 2100 cm^–1^ totally disappear, the concomitant appearance of a N=N stretching vibration peak around 1607 cm^–1^ demonstrates the formation of a 1,2,3-triazolyl linkage.^46^–^48^ The solid state ^13^C NMR spectra further indicate the presence of a 1,2,3-triazolyl linkage by the resonance of the C4-triazolyl carbon at 148 ppm (Fig. S2†).^11^,^46^ The broad signals at 145–120 and 65 ppm correspond to the aromatic carbon atoms and the central carbon of the tetraphenyl-methane core, respectively.^32^,^49^ The peak at 36 ppm is assigned to the 9-positioned carbon atom of the fluorene unit in POF-1, while the peak in POF-2 and POF-3 is shifted to 54 and 56 ppm, respectively, owing to the introduction of the propyl and benzyl substituents. The other peaks between 58–13 ppm in POF-2 and POF-3 may be ascribed to the alkyl carbon atoms of the substituents. In the TGA curves of POF-1, POF-2 and POF-3, initial weight losses of 5.3, 5.1 and 2.0% before 115 °C were observed, respectively (Fig. S3†). XRD analyses reveal they are amorphous due to kinetic irreversibility of the click reaction (Fig. S4†).^50^ Granular morphologies with particle diameters of 20–50 nm were observed in their SEM images (Fig. S5†).

**Fig. 1:**
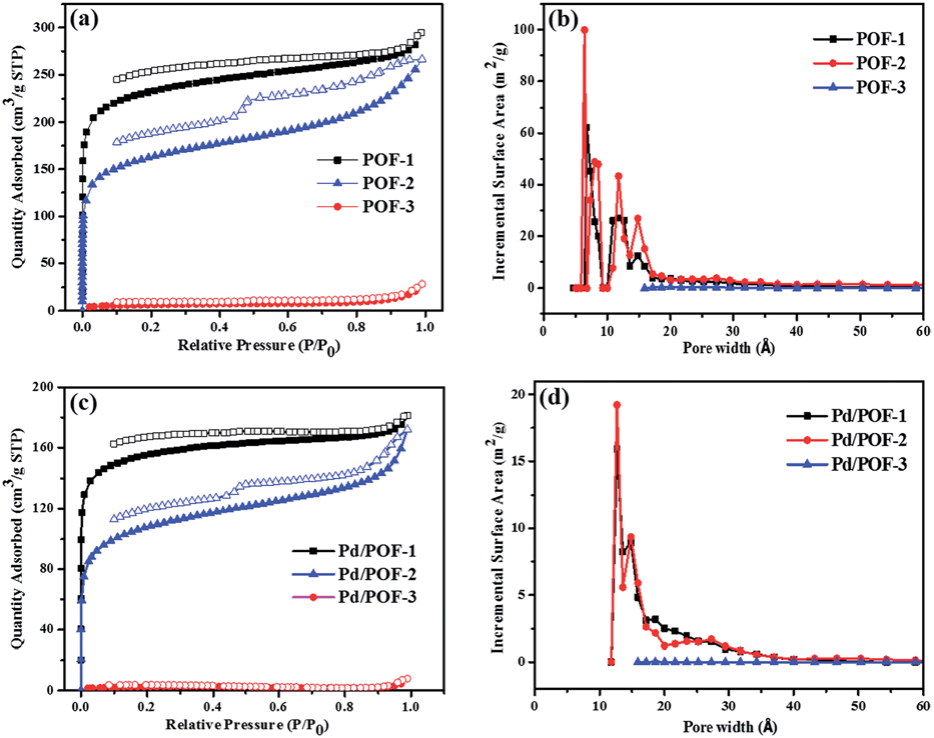
Nitrogen adsorption/desorption isotherms (a and c) and pore size distribution (b and d) for POF-1, POF-2, POF-3, Pd/POF-1, Pd/POF-2 and Pd/POF-3.

The porosities of POF-1, POF-2 and POF-3 were investigated by physisorption of nitrogen at 77 K (Fig. 1a). The rapid nitrogen uptake at very low relative pressure (*P*/*P*_0_ < 0.01) suggests the presence of extensive micropores in POF-1 and POF-2, while the uptake in POF-3 is negligible due to pore filling by the bulky benzyl groups.^51^–^53^ An obvious hysteresis and step (*P*/*P*_0_ = 0.46) were observed in the desorption isotherm of POF-2, owing to the interspersion of the propyl groups in the micropores.^26^ The BET surface area and total pore volume in POF-1 are 871 m^2^ g^–1^ and 0.43 cm^3^ g^–1^, respectively, which decrease to 622 m^2^ g^–1^ and 0.30 cm^3^ g^–1^, respectively, in POF-2 (Table S1†). The pore size distribution reveals that the predominant pores in POF-1 and POF-2 are in the microporous range (Fig. 1b). In contrast, POF-3 shows a negligible BET surface area of 23 m^2^ g^–1^ and no significant micropores were detected because the two benzyl groups at the 9-positions of fluorene units leads to the loss of the pores. The H_2_ adsorption capacities were also investigated at 77 K and 1 bar. As shown in Fig. S6a,† POF-1 and POF-2 exhibit high H_2_ uptakes of 115 and 103 cm^3^ g^–1^, respectively, due to their dominant microporous structure.^54^ Interestingly, the H_2_ uptake of POF-3 is 31 cm^3^ g^–1^ despite its negligible BET surface area. In addition, an obvious hysteresis was observed for POF-3.

Considering the important role of the substituents in modulating the porous structures of POFs, their influence on the palladium NPs were further investigated. The treatment of POF-1, POF-2 and POF-3 with Pd(OAc)_2_ in a 2 : 1 molar ratio of triazolyl to palladium in CH_2_Cl_2_ and subsequent reduction in a stream of H_2_/N_2_ gave rise to Pd/POF-1, Pd/POF-2 and Pd/POF-3, respectively. The palladium content in Pd/POF-1, Pd/POF-2 and Pd/POF-3 is 0.31, 0.43 and 0.61 mmol g^–1^, respectively. Notably, when the amount of Pd(OAc)_2_ is doubled for POF-1 under other identical conditions, the palladium content (0.33 mmol g^–1^) is similar to that in Pd/POF-1, which suggests that palladium loading is governed by the porosities of the POFs. The FTIR (Fig. S1†), solid-state ^13^C NMR (Fig. S2†) and SEM (Fig. S5†) results of Pd/POF-1, Pd/POF-2 and Pd/POF-3 are almost identical with those of POF-1, POF-2 and POF-3, respectively, indicating their structural frameworks are well maintained after the palladium loading.^11^ The thermal stabilities of Pd/POF-1, Pd/POF-2 and Pd/POF-3 are lower than those of POF-1, POF-2 and POF-3, respectively (Fig. S3†). In their XRD patterns, no obvious characteristic peaks of the palladium NPs were observed (Fig. S4†). The shapes of the N_2_ adsorption/desorption isotherms (Fig. 1c) in Pd/POF-1, Pd/POF-2 and Pd/POF-3 are preserved in comparison with their original frameworks, indicating that the pore systems have not been altered substantially after supporting the palladium NPs.^8^ The BET surface areas of Pd/POF-1, Pd/POF-2 and Pd/POF-3 decrease to 588, 438 and 9 m^2^ g^–1^, respectively, owing to partial pore filling and/or mass increment after palladium loading. It should be mentioned that the pore sizes less than 10 Å almost disappear in Pd/POF-1 and Pd/POF-2 (Fig. 1d). The H_2_ uptake amounts of Pd/POF-1, Pd/POF-2 and Pd/POF-3 are slightly decreased to 104, 90 and 28 cm^3^ g^–1^, respectively (Fig. S6b†).

**Fig. 2:**
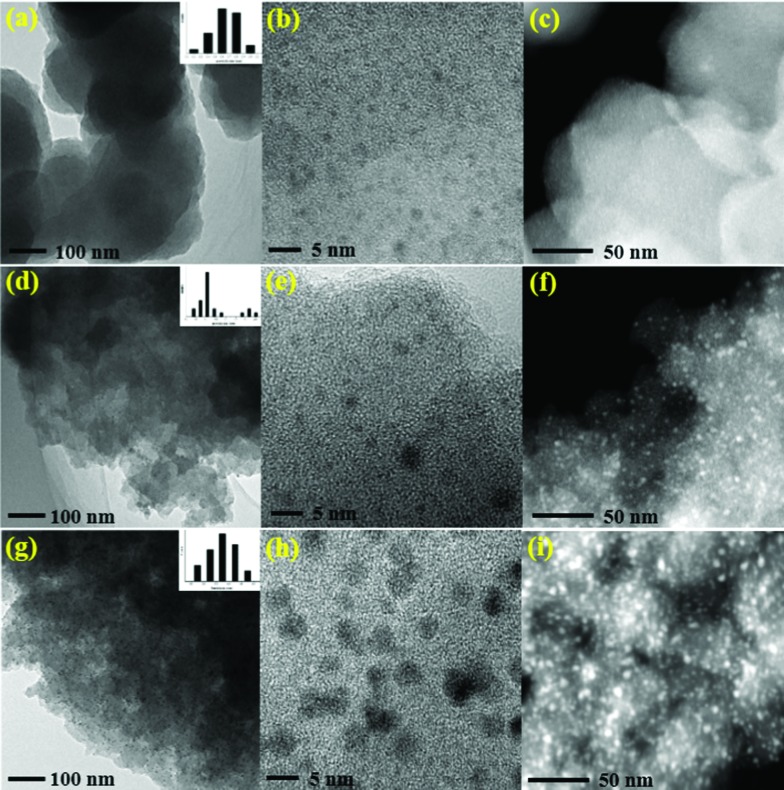
TEM and HAADF-STEM images of palladium NPs for Pd/POF-1 (a–c), Pd/POF-2 (d–f), and Pd/POF-3 (g–i).

Transmission electron microscope (TEM) images clearly show that the ultrafine palladium NPs in Pd/POF-1 are uniformly encapsulated in the interior pores of POF-1, and their average size is 1.60 ± 0.40 nm (Fig. 2a–c), which is small enough to be accommodated by the interior cavities of POF-1. In contrast, palladium NPs in Pd/POF-2 exhibit a dual size distribution. The relatively small palladium NPs with an average diameter of 2.15 ± 0.45 nm are located in the interior pores of POF-2, while large NPs with an average diameter of 3.65 ± 0.45 nm are deposited on the external surface of POF-2 (Fig. 2d–f). Interestingly, palladium NPs in Pd/POF-3 are uniformly dispersed on the external surface of POF-3 due to the absence of micropores, which are stabilized by both the coordination interaction of the surface 1,2,3-triazolyl linkages and the π interactions of the flexible benzyl groups. The average size of the palladium NPs in Pd/POF-3 is 3.55 ± 0.65 nm (Fig. 2g–i), which is close to that on the external surface of POF-2. HAADF-STEM further confirms the size, location and distribution of the palladium NPs in Pd/POF-1, Pd/POF-2 and Pd/POF-3 (Fig. 2c, f and i).

It has been reported that the amount of palladium precursors used may affect the size, location and distribution of the palladium NPs,^55^,^56^ however, no significant change in the TEM images of Pd/POF-1 was observed when two times the amount of Pd(OAc)_2_ was used under the same conditions (Fig. S7†), which further indicates that the palladium NPs are closely associated with the pore nature of the POFs.

**Fig. 3:**
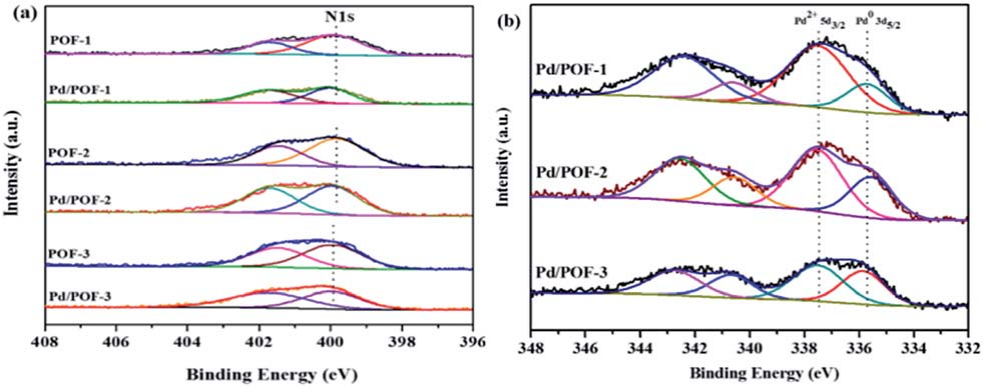
N 1s XPS spectra for (a) POF-1, POF-2, POF-3, Pd/POF-1, Pd/POF-2 and Pd/POF-3; (b) Pd 3d XPS spectra of Pd/POF-1, Pd/POF-2 and Pd/POF-3.

The X-ray photoelectron spectroscopy (XPS) results of POF-1, POF-2 and POF-3 are shown in Fig. S8.† The binding energy peaks at 284.8 and 399.8 eV correspond to C 1s and N 1s, respectively. The N 1s region is divided into two peaks, the peak at 401.5 eV corresponds to the N2 atom of the 1,2,3-triazolyl linkage, the other peak at 399.8 eV may be assigned to the N1 and N3 atoms, with the ratio of their relative peak areas being ∼1 : 2 (Fig. 3a), which further confirms the formation of the 1,2,3-triazolyl linkage.^50^ In order to clarify the interaction between the supports and the incorporated palladium species, the N 1s XPS spectra of Pd/POF-1, Pd/POF-2 and Pd/POF-3 were investigated (Fig. 3a). The binding energy peaks of the N 1s positively shift by 0.35, 0.25 and 0.05 eV, respectively, in comparison with their respective POFs, indicating that the interaction between the supports and the palladium NPs gradually weakens when the substituents change from hydrogen to propyl to benzyl. The existing states of the surface palladium in Pd/POFs were also investigated by XPS (Fig. 3b). The Pd 3d region is divided into two spin–orbital pairs, indicating that there are two types of the surface-bound palladium species.^7^ For Pd/POF-1, the binding energy peaks at 335.75 (Pd 3d_5/2_) and 340.65 eV (Pd 3d_3/2_) are assigned to Pd(0) species, while the peaks at 337.55 (Pd 3d_5/2_) and 342.45 eV (Pd 3d_3/2_) correspond to Pd(ii) species. The presence of Pd(ii) species is probably ascribed to the residual palladium acetate and/or reoxidation of Pd(0) during air contact.^50^ A comparison of the relative peak areas of the Pd(0) and Pd(ii) shows that the ratio of Pd(0) to Pd(ii) is 0.31. The Pd 3d_5/2_ binding energy peaks of the Pd(0) species in Pd/POF-2 and Pd/POF-3 are 335.60 eV and 336.05, respectively. In comparison with that in Pd/POF-1, the negative shift of 0.15 eV in Pd/POF-2 and positive shift of 0.30 eV in Pd/POF-3 may be ascribed to the inductive effect of the electron-donating propyl and electron-withdrawing benzyl, respectively, resulting in more electron-rich Pd(0) species in Pd/POF-2 and electron-deficient Pd(0) species in Pd/POF-3. The ratios of Pd(0) to Pd(ii) in Pd/POF-2 and Pd/POF-3 are 0.56 and 0.78, respectively. The gradually increased ratios of Pd(0) to Pd(ii) are consistent with the distribution trends of palladium NPs from the interior pores to the external surface. These observations further reveal that the porous nature of POFs has an important influence on the palladium NPs.

To have a clear insight into the influence of the substituents on the electronic properties of the POFs and the interactions between the POFs and palladium NPs, density functional theory (DFT) calculations were performed based on the POFs and Pd/POFs model compounds. As shown in Table S2,† the hydrogen, propyl and benzyl substituents of the fluorene units have a negligible influence on the bond distances, but they do slightly affect the dihedral angles of N2–N1–N4–N5 (*β*) and the angles between the pseudo-planes of the Pd/POFs (*τ*). The nitrogen atoms of the 1,2,3-triazolyl in both the POFs and Pd/POFs possess negative charges, but the N1 atom is more negative than the N2 and N3. This demonstrates that N1 possesses more charge density and prefers to coordinate with palladium (Fig. S9†), which is the same as those in click-based coordination compounds.^8^ Notably, the charge of N1 in the Pd/POFs is more negative than that in the POFs, while the charges of N2 and N3 in the Pd/POFs are more positive than those in the POFs, indicating that the electrons transfer from N2 and N3 to N1 after the palladium loading. The charges of the fluorene units in POF-1 and Pd/POF-1 are 0.00373 and 0.00363*e*, respectively. The introduction of the propyl and benzyl substituents result in slightly negative and positive shifts, respectively (Table S3†), which are consistent with the results of the XPS analysis. The highest occupied molecular orbital (HOMO) and the lowest unoccupied molecular orbital (LUMO) in the POFs are shared by the triazolyl and fluorene units owing to their conjugative effect. However, the HOMOs in the Pd/POFs are mainly occupied by the palladium species, and the LUMOs are shared by the triazolyl and fluorene units (Fig. S10†).

**Fig. 4:**
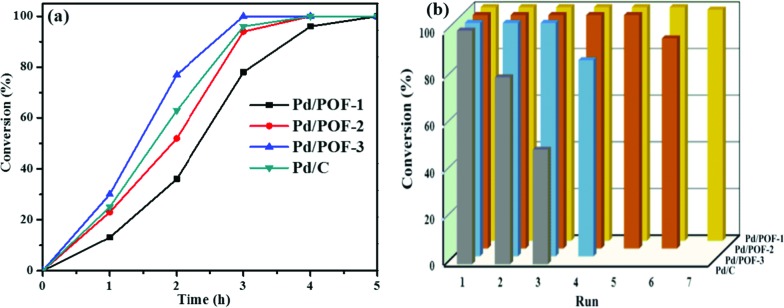
(a) The conversion of styrene as a function of time in hydrogenation reactions. (b) Recyclability of Pd/POF-1, Pd/POF-2, Pd/POF-3 and Pd/C. Reaction conditions: styrene (2 mL, 21 mmol), [Pd] (0.02 mol%), 1.0 atm of H_2_, 25 °C, 6 h.

To understand the roles of the size, location and distribution of palladium NPs in catalytic reactions, Pd/POF-1, Pd/POF-2 and Pd/POF-3 were initially evaluated by the solvent-free hydrogenation of styrene under 25 °C and 1.0 atm H_2_. As shown in Fig. 4a, Pd/POF-1 afforded a full conversion of styrene to phenylethane in 5 h, while the use of Pd/POF-3 gave a complete conversion in 3 h under the same conditions. As expected, the catalytic activity of Pd/POF-2 was in the middle of Pd/POF-1 and Pd/POF-3 owing to the dual size distribution of palladium NPs in the interior pores and on the external surface (Fig. 2). The highest catalytic activity of Pd/POF-3 is probably attributed to the improvement of mass transport and easy availability of active sites on the external surface. As a comparison, commercial Pd/C was also tested under the same conditions and a complete conversion of styrene was achieved in 4 h, which is inferior to that of Pd/POF-3.

Besides activity, other important factors for a heterogeneous catalytic system, such as recyclability and stability, were also examined. As shown in Fig. 4b, Pd/POF-1 could be used for at least 7 runs with 100% conversion of styrene, while conversions of 90 and 83% were achieved after the hydrogenation reactions of Pd/POF-2 and Pd/POF-3 were performed for six and four runs, respectively. In contrast, Pd/C afforded 80 and 49% conversion of styrene in the second and third runs, respectively.

**Fig. 5:**
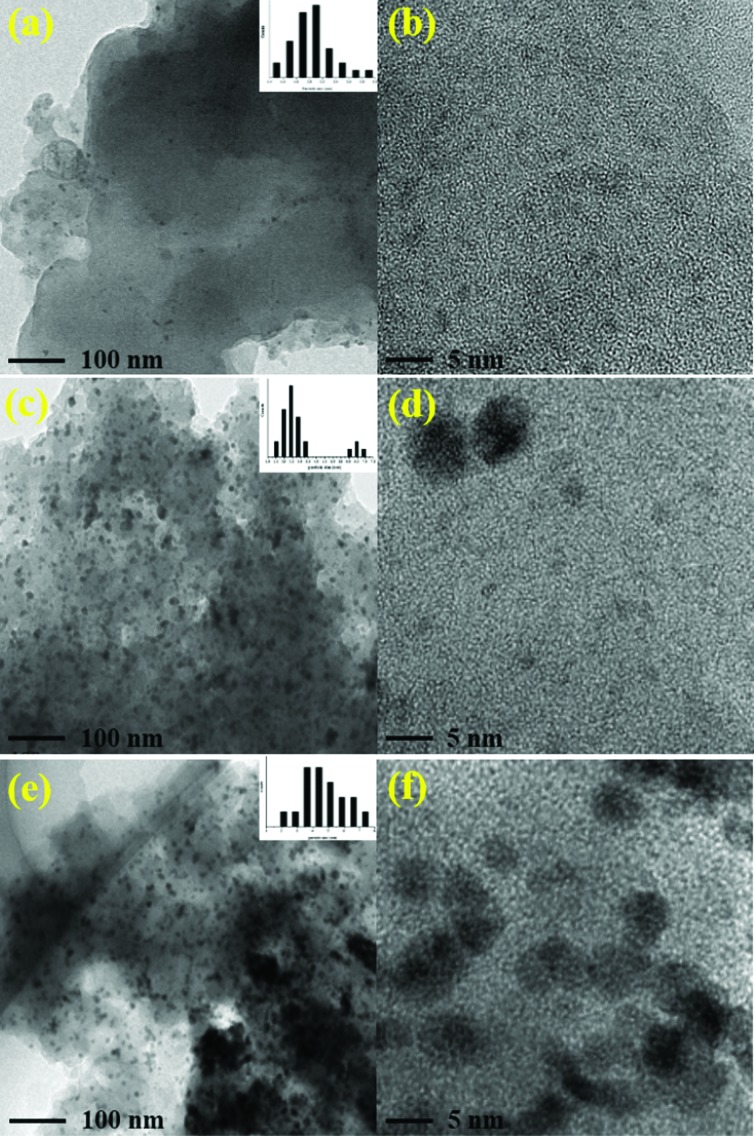
TEM images for Pd/POF-1-run7 (a and b), Pd/POF-2-run6 (c and d) and Pd/POF-3-run4 (e and f).

To explore the stability of palladium NPs in these catalytic systems, the black powders were isolated after consecutive reactions and examined by TEM. As shown in Fig. 5, the majority of the palladium NPs in Pd/POF-1-run7 are in the interior pores of the host framework, with the average size of palladium NPs slightly increased to 2.0 nm. However, a few palladium NPs diffuse out the micropores and aggregate on the external surface of POF-1 (Fig. 5a and b), which is consistent with the reported NPs encapsulated in the interior pores of POFs.^8^,^11^ Interestingly, the palladium NPs in Pd/POF-2-run6 maintain their dual size distribution, the average size of palladium NPs in the interior pores and the external surface are increased from 2.15 to 2.50 nm and from 3.65 to 6.50 nm, respectively (Fig. 5c and d). For Pd/POF-3, an obvious agglomeration was observed after the fourth run (Fig. 5e and f). The superior stability of the palladium NPs in the interior cavities of POF-1 probably result from the confinement interaction of the host framework and the coordination interaction of 1,2,3-triazolyl linkage. However, the coordination interaction of the 1,2,3-triazolyl and the π interaction of benzyl ring are not enough to efficiently immobilize the palladium NPs on the external surface of POF-3, resulting in deactivation and the loss of catalytic activity after consecutive reactions. ICP analyses demonstrate that the palladium leaching after the first run of hydrogenation for Pd/POF-1, Pd/POF-2 and Pd/POF-3 is 0.76, 1.41 and 2.09 ppm, respectively, which is agreement with the stability of palladium NPs.

The stability of Pd/POF-1, Pd/POF-2 and Pd/POF-3 were also examined after they were stored in air over half a year. As shown in Fig. S11,† the size, location and distribution of palladium NPs in Pd/POF-1 have no significant change in comparison with the fresh sample, however, the average size of palladium NPs on the external surface of Pd/POF-2 increases to 4.50 nm and an obvious agglomeration of palladium NPs was observed in Pd/POF-3. This further reveals that palladium NPs encapsulated in the interior pores of POFs possess a higher stability than those deposited on the external surface.

The outstanding catalytic recyclability and stability of Pd/POF-1 for the hydrogenation of styrene encouraged us to explore the generality of the catalytic system. As shown in Table 1, the hydrogenation of styrene afforded a 100% GC yield under 1.0 atm H_2_ at 25 °C for 6 h (entry 1), while no target product was detected when the control experiment was performed in the presence of only POF-1 (entry 2). When Pd(OAc)_2_ supported by POF-1 was used as a catalyst, only a trace amount of phenylethane was formed (entry 3). A series of chain-, cyclo- and phenyl-olefins were also tested under the same conditions. The use of linear 1-hexene and 1-octene generated the corresponding products in quantitative yields in 6 h (entries 4 and 5). Interestingly, the hydrogenation of cyclohexene also gave rise to the target product in a quantitative yield in 6 h (entry 6). The catalytic system was also effective for the hydrogenation of 1,4-cyclohexadiene. The target product was obtained in 100% conversion and 77% cyclohexane selectivity in 6 h (entry 7), complete hydrogenation to cyclohexane was achieved when the reaction time was prolonged to 12 h (entry 8). Notably, after the hydrogenation of styrene was run for 1 h, the palladium-containing catalytic species were quickly removed by filtration, and the filtrate continued to react for the additional 11 h, a negligible change in conversion was observed (entries 9 and 10) indicating that the hydrogenation proceeds in a heterogeneous manner in the catalytic system. The catalytic activity of palladium NPs was also examined after they were stored in air over half a year. In comparison with fresh samples, no significant change was observed for the stored Pd/POF-1 and Pd/POF-2 (entries 11 and 12). However, the conversion for the stored Pd/POF-3 decreased to 86% (entry 13).

**Table 1:** Hydrogenation of olefins^*a*^

Entry	Olefins	Time (h)	Con.^*b*^ (%)	Sel.^*b*^ (%)
1	Styrene	6	100	100
2^*c*^	Styrene	6	0	0
3^*d*^	Styrene	6	<3	100
4	1-Hexene	6	100	100
5	1-Octene	6	100	100
6	Cyclohexene	6	100	100
7	1,4-Cyclohexadiene	6	100	77
8	1,4-Cyclohexadiene	12	100	100
9	Styrene	1	15	100
10^*e*^	—	12	15	100
11^*f*^	Styrene	6	100	100
12^*g*^	Styrene	6	100	100
13^*h*^	Styrene	6	86	100

^*a*^Hydrogenation was performed in olefins (2 mL) and [olefins (mol)/Pd (mol) = 5000] in 1.0 atm H_2_ at 25 °C.

^*b*^Conversion and selectivity were determined by GC.

^*c*^POF-1 was used in the absence of palladium.

^*d*^Pd(OAc)_2_/POF-1 was used as a catalyst.

^*e*^Filtration experiment.

^*f*^Pd/POF-1 was stored over half a year.

^*g*^Pd/POF-2 was stored over half a year.

^*h*^Pd/POF-3 was stored over half a year.

## Conclusions

The porosities of POFs were modulated through varying coordination-inert substituents of fluorene-based building units at the molecular level. These POFs can serve as effective supports for the synthesis of palladium NPs with a tunable size, location and distribution by a substituent-controlled strategy. To our knowledge, this is the first report for the synthesis of metal NPs through tailor-made porous properties of POFs. The surface area, pore volume and pore size in the POFs have exerted important influences on the palladium NPs and their catalytic performances. In Pd/POF-1, Pd/POF-2 and Pd/POF-3, the coordination interaction between the supports and the palladium NPs gradually weakens with a concomitant increase of the Pd(0) to Pd(ii) ratios. Pd/POF-3, with palladium NPs on the external surface, shows the highest catalytic activity in the hydrogenation of olefins because of the improvement of mass transport and easy availability of active sites, while Pd/POF-1, with ultrafine palladium NPs in the interior pores, exhibits the highest stability and recyclability owing to the confinement effect of the host framework and the coordination interaction of the 1,2,3-triazolyl linkage. In summary, this study has established an appealing platform for the pre-designable fabrication of metal NPs with size, location and distribution control using a substituent-controlled strategy, which opens a new and general avenue for the synthesis of metal NPs immobilized by POFs. Further study will focus on the tunable synthesis of metal NPs with specific performances through molecular functionalization of modular building units in POFs.

## Supplementary Material

Supplementary information
