# Supplementary material for: Tailor-made porosities of fluorene-based porous organic frameworks for the pre-designable fabrication of palladium nanoparticles with size, location and distribution control
Source: Chem Sci. 2015 Dec 10;7(3):2188–94. doi: 10.1039/c5sc04351d (PMC5975943; doi:10.1039/c5sc04351d)
Supplement: Supplementary file 1 [file SC-007-C5SC04351D-s001.pdf]

Electronic Supplementary Material (ESI) for Chemical Science.
This journal is © The Royal Society of Chemistry 2015

Supporting information

for

Tailor-made Porosities of Fluorene-based Porous Organic Frameworks for Pre-designable Fabrication of Palladium Nanoparticles with Size, Location and Distribution Control

Hong Zhong, Caiping Liu, Yangxin Wang, Ruihu Wang, Maochun Hong*

Methods

Materials and characterization. 2,7-diethynylfluorene,[1] 2,7-diethynyl-9,9-dipropylfluorene,[1] 2,7-diethynyl-9,9-dibenzylfluorene[1] and tetrakis(4-azidophenyl)methane[2,3] were prepared according to the modified literature methods. Other chemicals were commercially available and used without further purification. ^{1}H and ^{13}C NMR spectra were recorded on a Bruker AVANCE III NMR spectrometer at 400 and 100 MHz, respectively, using tetramethylsilane (TMS) as an internal standard and $CDCl_3$ as a locking solvent. Solid-state ^{13}C CP/MAS NMR was performed on a Bruker SB Avance III 500 MHz spectrometer with a 4-mm double-resonance MAS probe, a sample spinning rate of 7.0 kHz, a contact time of 2 ms and pulse delay of 5 s. FTIR spectra were recorded with KBr pellets using Perkin-Elmer Instrument. Thermal gravimetric analysis (TGA) was carried out on NETZSCH STA 449C by heating samples from 30 to 800 ^{o}C in a dynamic nitrogen atmosphere with a heating rate of 10 $°C \cdot min^{-1}$. Powered X-ray diffraction (XRD) patterns were recorded in the range of $2\theta = 5\text{-}85^{o}$ on a desktop X-ray diffractometer (RIGAKU-Miniflex II) with Cu K_{α} radiation ($\lambda = 1.5406$ Å). Adsorption and desorption isotherms of nitrogen and hydrogen were measured at 77 K using a Micromeritics ASAP 2020 system. The samples were degassed at 100 ^{o}C for 10 h before the measurements. Surface areas were calculated from the adsorption data using Brunauer-Emmett-Teller (BET) and Langmuir methods. The pore size distribution curves were obtained from the adsorption branches using non-local density functional theory (NLDFT) method. Field-emission scanning electron microscopy (SEM) was performed on a JEOL JSM-7500F operated at an accelerating voltage of 3.0 kV. Transmission electron microscope (TEM) images were obtained with a JEOL JEM-2010 instrument operated at 200 kV. X-ray photoelectron spectroscopy (XPS) measurements were performed on a Thermo ESCALAB 250 spectrometer, using non-monochromatic Al K_{α} X-rays as the excitation source and choosing C 1s (284.6 eV) as the reference line. Inductively coupled plasma (ICP) measurement was performed on Jobin Yvon Ultima 2. Gas chromatography (GC) was performed on a Shimadzu GC-2014 equipped with a

capillary column (RTX-5, 30 m × 0.25 μm) using a flame ionization detector. Elemental analyses were performed on an Elementar Vario MICRO Elemental analyzer.

Synthesis of 2,7-di(trimethylsilylethynyl)fluorene. 2,7-Dibromofluorene (1.62 g, 5.0 mmol), trimethylsilylacetylene (4.90 g, 50 mmol), $Pd(PPh_3)_4$ (0.58g, 0.50 mmol) and CuI (0.10 g, 0.50 mmol) were added into a mixture of THF (30 mL) and diisopropylamine (10 mL) under nitrogen, the reaction mixture was stirred at 75 ^{o}C for 24 h. After cooled to room temperature, the resulting suspension was filtered with celite, the filtrate was successively washed with 10 % aqueous HCl (30 mL x 3), H_2O (30 mL x 3), saturated $NaHCO_3$ (30 mL x 3), H_2O (30 mL x 3), and dried using anhydrous $MgSO_4$. Subsequent concentration by rotary evaporation gave a crude product, which was purified by flash column chromatography on silica gel to afford pale yellow solids. Yield: 0.95 g, (53%). ^{1}H NMR: δ 7.70 (d, J = 7.90 Hz, 2H), 7.66 (s, 2H), 7.51 (d, J = 7.90 Hz, 2H), 3.87 (s, 2H), 0.29 (s, 18H). ^{13}C NMR: δ 143.4, 141.3, 131.0, 128.6, 121.6, 112.0, 105.7, 94.5, 36.5, 0.1 ppm. FTIR (KBr, cm^{-1}): 3036 (w), 2960 (m), 2898 (m), 2147 (m), 1466 (m), 1415 (m), 1250 (m), 930 (m), 841 (s), 761 (m), 705 (w), 646 (m).

Synthesis of 2,7-diethynylfluorene. 2,7-Di(trimethylsilylethynyl)fluorene (1.07 g, 3.0 mmol) and K_2CO_3 (2.07 g 15.0 mmol) were added to THF/CH_3OH (20/20 mL), the mixture was stirred at room temperature overnight. The resulting suspension was filtered and concentrated by rotary evaporation. Column chromatography on silica gel afforded the target products. Yield: 0.55 g, (86%). ^{1}H NMR: δ 7.74 (d, J = 7.88 Hz, 2H), 7.70 (s, 2H), 7.55 (d, J = 7.88 Hz, 2H), 3.91 (s, 2H), 3.15 (s, 2H). ^{13}C NMR: δ 143.5, 141.5, 131.1, 128.8, 120.7, 120.0, 84.2, 77.2, 37.5 ppm. FTIR (KBr): 3280 (s), 3037 (w), 2916 (w), 2098 (m), 1610 (m), 1465 (m), 1415 (m), 1280 (m), 939 (w), 825 (s), 675 (m), 625 (m).

Synthesis of 2,7-dibromo-9,9-dipropylfluorene. 2,7-Dibromofluorene (3.24 g, 10.0 mmol) and TBAB (0.32 g 1.0 mmol) were added into 50 % aqueous NaOH (50 mL) under nitrogen. After the mixture was stirred at 70 ^{o}C for 0.5 h, 1-bromopropane

(7.38 g, 60.0 mmol) was added to the suspension by syringe and stirred at 70 ^oC for 24 h. The resulting mixture was cooled to room temperature and extracted with CH_2Cl_2 (20 mL x 3). The organic extracts were washed with H_2O (20 mL x 3), saturated brine (20 mL x 3), and dried using anhydrous $MgSO_4$. After filtering, the filtrate was concentrated by rotary evaporation to give a crude product, which was purified by recrystallize in ethanol to afford white products. Yield: 3.06 g, (75%). ^1H NMR: δ 7.54 (d, J = 8.7 Hz, 2H), 7.48 (s, 2H), 7.47 (d, J = 8.7 Hz, 2H), 1.93 (t, J = 7.0 Hz, 4H), 0.65 - 0.74 (m, 10H). ^{13}C NMR: δ 152.6, 139.1, 130.2, 126.2, 121.5, 121.1, 55.9, 42.5, 17.1, 14.3 ppm. FTIR (KBr): 3078 (w), 3052 (w), 2948(s), 2925(s), 2899(s), 2839 (m), 1662 (m), 1598 (m), 1449 (s), 1415 (s), 1397 (m), 1378 (m), 1056 (s), 930 (m), 875 (m), 807 (s), 746 (s).

Synthesis of 2,7-di(trimethylsilylethynyl)-9,9-dipropylfluorene. The complex was prepared using the similar method to that of 2,7-di(trimethylsilylethynyl)fluorene except that 2,7-Dibromofluorene was replaced by 2,7-dibromo-9,9-dipropylfluorene Yield: 61%. ^1H NMR: δ 7.61 (d, J = 7.8 Hz, 2H), 7.47 (d, J = 7.8 Hz, 2H), 7.45 (s, 2H), 1.95 (t, J = 7.6 Hz, 4H), 0.58 - 0.70 (m, 10H), 0.31 (s, 18H). ^{13}C NMR: δ 151.0, 140.6, 131.2, 126.3, 121.8, 119.8, 106.0, 94.3, 55.4, 42.6, 17.0, 14.3, 0.1 ppm. FTIR (KBr): 3029 (w), 2953 (m), 2897 (m), 2157 (m), 1463 (m), 1422 (m), 1381 (w), 1247 (m), 955 (m), 880 (s), 840 (s), 761 (m), 647 (m).

Synthesis of 2,7-diethynyl-9,9-dipropylfluorene. The complex was prepared using similar method to 2,7-diethynylfluorene except that 2,7-di(trimethylsilylethynyl)fluorene was replaced by 2,7-di(trimethylsilylethynyl)-9,9-dipropylfluorene. Yield: 89%. ^1H NMR: δ 7.66 (s, 1H), 7.64 (d, J = 8.2 Hz, 2H), 7.51 (d, J = 8.2 Hz, 2H), 7.49 (2, 2H), 3.17 (s, 2H), 1.95 (t, J = 7.1 Hz, 4H), 0.63 - 0.70 (m, 10H). ^{13}C NMR: δ 151.1, 141.0, 131.3, 126.6, 120.9, 120.0, 84.5, 77.2, 55.4, 42.5, 17.1, 14.3 ppm. FTIR (KBr): 3280 (s), 3069 (w), 3024 (w), 2956 (s), 2931 (s), 2870 (m), 2104 (m), 1463 (m), 1419 (m), 1376 (w), 1203 (m), 826 (s), 757 (w), 653 (s), 627 (s).

Synthesis of 2,7-dibromo-9,9-dibenzylfluorene. The complex was prepared using similar method to 2,7-dibromo-9,9-dipropylfluorene except that 1-bromopropane was replaced by bromomethylbenzene. Yield: 78 %. ^{1}H NMR: δ 7.53 (d, J = 1.8 Hz, 2H), 7.37 (d, J = 8.1 Hz, 2H), 7.23 (d, J = 8.1 Hz, 2H), 6.98-7.04 (m, 6H), 6.69 (d, J = 7.6 Hz, 4H), 3.35 (s, 4H). ^{13}C NMR: δ 150.2, 138.8, 136.1, 130.3, 130.1, 128.0, 127.4, 126.3, 121.1, 120.6, 57.2, 45.2 ppm. IR (KBr): 3023 (m), 2914 (m), 2853 (m), 1599 (m), 1493 (m), 1448 (s), 1395 (m), 1261 (m), 1061 (m), 881 (m), 812 (s), 699 (s), 689 (m).

Synthesis of 2,7-di(trimethylsilylethynyl)-9,9-dibenzylfluorene. The complex was prepared using the similar method to that of 2,7-di(trimethylsilylethynyl)fluorene except that 2,7-Dibromofluorene was replaced by 2,7-dibromo-9,9-dibenzylfluorene. Yield: 67%. ^{1}H NMR: δ 7.56 (s, 2H), 7.35 (d, J = 7.8 Hz, 2H), 7.28 (d, J = 7.8 Hz, 2H), 6.92 - 7.01 (m, 6H), 6.67 (d, J = 8.0 Hz, 4H), 3.39 (s, 4H), 0.34 (s, 18H). ^{13}C NMR: δ 148.5, 140.6, 136.4, 131.4, 130.2, 128.0, 127.2, 126.0, 121.2, 119.8, 106.0, 94.3, 56.9, 45.3, 29.7 ppm. FTIR (KBr): 3028 (w), 2924 (s), 2853 (s), 2156 (m), 1495 (m), 1463 (m), 1425 (m), 1248 (m), 891 (m), 846 (s), 760 (m), 697 (m), 645 (m).

Synthesis of 2,7-diethynyl-9,9-dibenzylfluorene. The complex was prepared using similar method to 2,7-diethynylfluorene except that 2,7-di(trimethylsilylethynyl)fluorene was replaced by 2,7-di(trimethylsilylethynyl)-9,9-dibenzylfluorene. Yield: 90%. ^{1}H NMR: δ 7.61 (s, 2H), 7.39 (d, J = 7.8 Hz, 2H), 7.32 (d, J = 7.8 Hz, 2H), 6.94-7.00 (m, 6H), 6.67 (d, J =8.1 Hz, 4H), 3.40 (s, 4H), 3.20 (s, 2H). ^{13}C NMR: δ 148.6, 140.8, 136.3, 131.5, 130.1, 128.3, 127.3, 126.1, 120.2, 112.0, 84.5, 77.2, 57.0, 45.3 ppm. FTIR (KBr): 3282 (s), 3028 (m), 2914 (m), 2847 (m), 2098 (m), 1599 (m), 1493 (m), 1463 (m), 827 (s), 736 (m), 700 (s), 619 (s), 572 (m).

Synthesis of tetrakis(4-azidophenyl)methane. Tetrakis(4-azidophenyl)methane was prepared according to the reported method. ^{1}H NMR: δ 7.15 (d, J = 8.7 Hz, 8H), 6.96 (d, J = 8.7 Hz, 8H). ^{13}C NMR: δ 142.9, 138.2, 132.1, 118.4, 63.2 ppm. FTIR (KBr): 3032 (w), 2931 (w), 2124 (s), 1602 (w), 1501 (s), 1290 (s), 829 (m).

Synthesis of POF-1. A mixture of 2,7-diethynylfluorene (0.43 g, 2.00 mmol), tetrakis(4-azidophenyl)methane (0.48 g, 0.10 mmol), $CuSO_4 \cdot 5H_2O$ (0.10 g, 0.40 mmol) and sodium ascorbate (0.16 g, 0.80 mmol) in dry DMF (30 mL) was stirred under nitrogen at 100 °C for 72 h to afford a yellow powder. The solid was isolated by filtration, and subsequently washed with aqueous EDTA-2Na solution (0.25 g in 200 mL H_2O), ethanol and CH_2Cl_2 to remove any unreacted monomers or residues. The yellow powder was further treated by Soxhlet extraction in THF overnight and dried *in vacuo* at 80 °C for 12 h. Yield: 873.6 mg (96 %). Elemental analysis calculated (%) for $C_{30}H_{18}N_6$: C 77.90, H 3.92, N 18.17. Found: C 67.67, H 4.87, N 15.43. FTIR (KBr): 3057 (w), 1607 (m), 1516 (s), 1416 (w), 1403 (m), 1224 (m), 1033 (s), 820 (s), 792 (w), 578 (w).

Synthesis of POF-2. POF-2 was prepared using similar method to POF-1 except that 2,7-diethynylfluorene was replaced by 2,7-diethynyl-9,9-dipropylfluorene. Yield: 98 %. Elemental analysis calculated (%) for $C_{36}H_{30}N_6$: C 79.12, H 5.49, N 15.38. Found: C 75.30, H 5.94, N 13.89. FTIR (KBr): 3052 (w), 2950 (m), 2865 (m), 1608 (m), 1509 (s), 1466 (m), 1224 (m), 1033 (s), 827 (s).

Synthesis of POF-3. POF-3 was prepared using similar method to POF-1 except that 2,7-diethynylfluorene was replaced by 2,7-diethynyl-9,9-dibenzylfluorene. Yield: 99%. Elemental analysis calculated (%) for $C_{44}H_{30}N_6$: C 82.24, H 4.67, N 13.08. Found: C 77.65, H 5.06, N 11.85. FTIR (KBr): 3064 (w), 3028 (w), 1608 (m), 1515 (s), 1403 (m), 1224 (m), 1033 (s), 826 (s).

Synthesis of Pd/POF-1. POF-1 (0.30 g) was added into a CH_2Cl_2 solution (300 mL) of palladium acetate (0.10 g, 0.44 mmol, N/Pd = 6), the mixture was vigorously stirred at 60 °C for 24 h. The resulting product was washed thoroughly with CH_2Cl_2 to remove excess of palladium acetate and dried *in vacuo* at 80 °C for 12 h. Subsequent reduction in a stream of 5 % H_2/N_2 at 200 °C for 4 h gave rise to gray Pd/POF-1. Yield: 311.5 mg (90%). FTIR (KBr): 3127 (m), 3049 (m), 1607 (m), 1508 (s), 1465 (m), 1408 (m), 1230 (m), 1038 (s), 819 (s).

Synthesis of Pd/POF-2. Pd/POF-2 was prepared using similar method to Pd/POF-1 except that POF-1 was replaced by POF-2. Yield: 89%. FTIR (KBr): 3134 (w), 3049 (w), 2957 (m), 2928 (m), 2871 (m), 1602 (m), 1508 (s), 1458 (m), 1401 (m), 1223 (m), 1032 (s), 819 (s).

Synthesis of Pd/POF-3. Pd/POF-3 was prepared using similar method to Pd/POF-1 except that POF-1 was replaced by POF-3. Yield: 89%. FTIR (KBr): 3067 (w), 3021 (w), 2921 (m), 2856 (m), 1602 (m), 1510 (s), 1460 (m), 1401 (m), 1224 (m), 1032 (s), 825 (s), 698 (s).

General procedures for hydrogenation of olefins. Hydrogenation was carried out in a high-pressure reactor equipped with a stirrer. A mixture of olefins (2 mL) and Pd/POFs (0.02 mmol% Pd) was stirred under 1.0 atm H_2 at 25 °C. After the reaction was completed, the product was removed from the reaction mixture with syringe. Conversion and selectivity were determined by GC, the identity of the products was confirmed by comparison with literature spectroscopic data.

General procedures for Recyclability test. After hydrogenation reaction of styrene in the presence of Pd/POFs under 1.0 atm H_2 at 25 °C was finished, the product was removed directly from the reaction mixture with syringe, conversion and selectivity were determined by GC. The remaining catalytic species were dried at 50 ^{o}C *in vacuo* and reused for the next run with the recharge of styrene.

Computational Methodology

The detailed density functional theory (DFT) study was performed to examine the electronic structures and corresponding properties of POFs and Pd/POFs model compounds. The hybrid functional of Becke three-parameter Lee Yang Parr (B3LYP)[3] was employed for all calculations, which showed a good performance to interpret the substituent effects and the interactions between POFs and palladium NPs.[4] Palladium atoms were modeled with the effective core potential and corresponding basis sets of Hey and Wadt (i.e. LANL2DZ ECP basis set)[5], and other non-metal atoms were modeled with the 6-31G(d) basis set as including single d polarization function for heavy atoms. All equilibrium geometries and electronic

properties of model compounds were calculated without any symmetric constraints in the gas phase. As for the natural population analyses, the calculations were performed by the NBO 3.1 module embedded in Gaussian 09 program[6]. The wavefunction analyses were performed by Multiwfn, which is a multifunctional wavefunction analysis program developed by Lu et. al. and can be freely downloaded[7]. During the calculation processes, the default settings were used for all programs. All isosurface maps including the frontier molecular orbitals and the electrostatic potential on van der Waals surface were rendered by VMD[8] and GaussView programs.

References:

1. D. J. V. C. Steenis, O. R. P. David, G. P. F. Strijdonck, J. H. Maarseveen and J. N. H. Reek, *Chem. Commun.*, 2005, 4333-4335.
2. M. Minato and P. M.Lahti, *J. Am. Chem. Soc.* **1997**, *119*, 2187-2195.
3. O. Plietzsch, C. I. Schilling, M. Tolev, M. Nieger, C. Richert, T. Muller, S. Brase, *Org. Biomol. Chem.*, 2009, **7**, 4734-4743.
4. (a) A. D. Becke, *J. Chem. Phys.* 1993, **98**, 5648. (b) P. J. Stephens, F. J. Devlin, C. F. Chabalowski and M. J. Frisch, *J. Phys. Chem.* 1994, **98**, 11623.
5. P. J. Hay and W. R. Wadt, *J. Chem. Phys.* 1985, **82**, 299.
6. M. J. Frisch, G. W. Trucks, H. B. Schlegel, G. E. Scuseria, M. A. Robb, J. R. Cheeseman, G. Scalmani, V. Barone, B. Mennucci, G. A. Petersson, H. Nakatsuji, M. Caricato, X. Li, H. P. Hratchian, A. F. Izmaylov, J. Bloino, G. Zheng, J. L. Sonnenberg, M. Hada, M. Ehara, K. Toyota, R. Fukuda, J. Hasegawa, M. Ishida, T. Nakajima, Y. Honda, O. Kitao, H. Nakai, T. Vreven, J. A. Montgomery, Jr., J. E. Peralta, F. Ogliaro, M. Bearpark, J. J. Heyd, E. Brothers, K. N. Kudin, V. N. Staroverov, R. Kobayashi, J. Normand, K. Raghavachari, A. Rendell, J. C. Burant, S. S. Iyengar, J. Tomasi, M. Cossi, N. Rega, J. M. Millam, M. Klene, J. E. Knox, J. B. Cross, V. Bakken, C. Adamo, J. Jaramillo, R. Gomperts, R. E. Stratmann, O. Yazyev, A. J. Austin, R. Cammi, C. Pomelli, J. W. Ochterski, R. L. Martin, K. Morokuma, V. G. Zakrzewski, G. A.

Voth, P. Salvador, J. J. Dannenberg, S. Dapprich, A. D. Daniels, Ö. Farkas, J. B. Foresman, J. V. Ortiz, J. Cioslowski and D. J. Fox, Gaussian, Inc., Wallingford CT, 2009.

7. T. Lu and F. Chen, *J. Comp. Chem.*, 2012, **33**, 580
8. W. Humphrey, A. Dalke and K. Schulten, *J Mol Graph.* 1996, **14**, 33

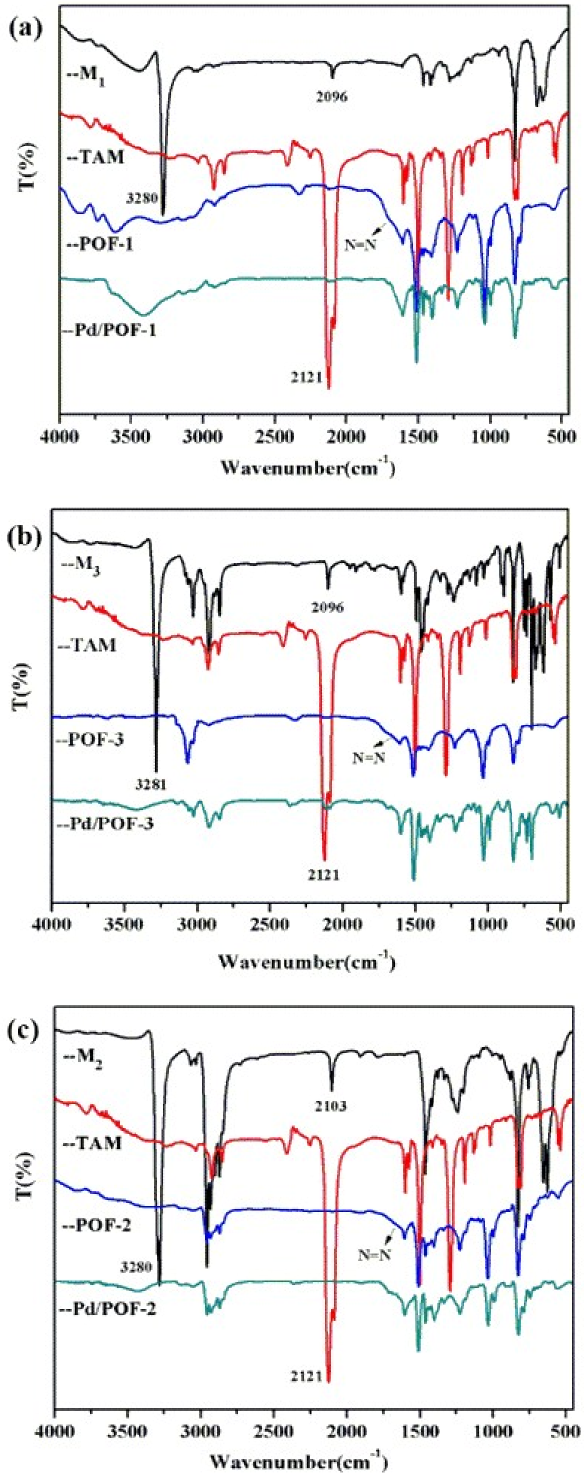

Figure S1. FTIR spectra for (a) starting materials, POF-1 and Pd/POF-1; (b) starting materials, POF-2 and Pd/POF-2; (c) starting materials, POF-3 and Pd/POF-3. (M1: 2,7-diethynylfluorene, M2: 2,7-diethynyl-9,9-dipropylfluorene, M3: 2,7-diethynyl-9,9-dibenzylfluorene, TAM: tetrakis(4-azidophenyl)methane).

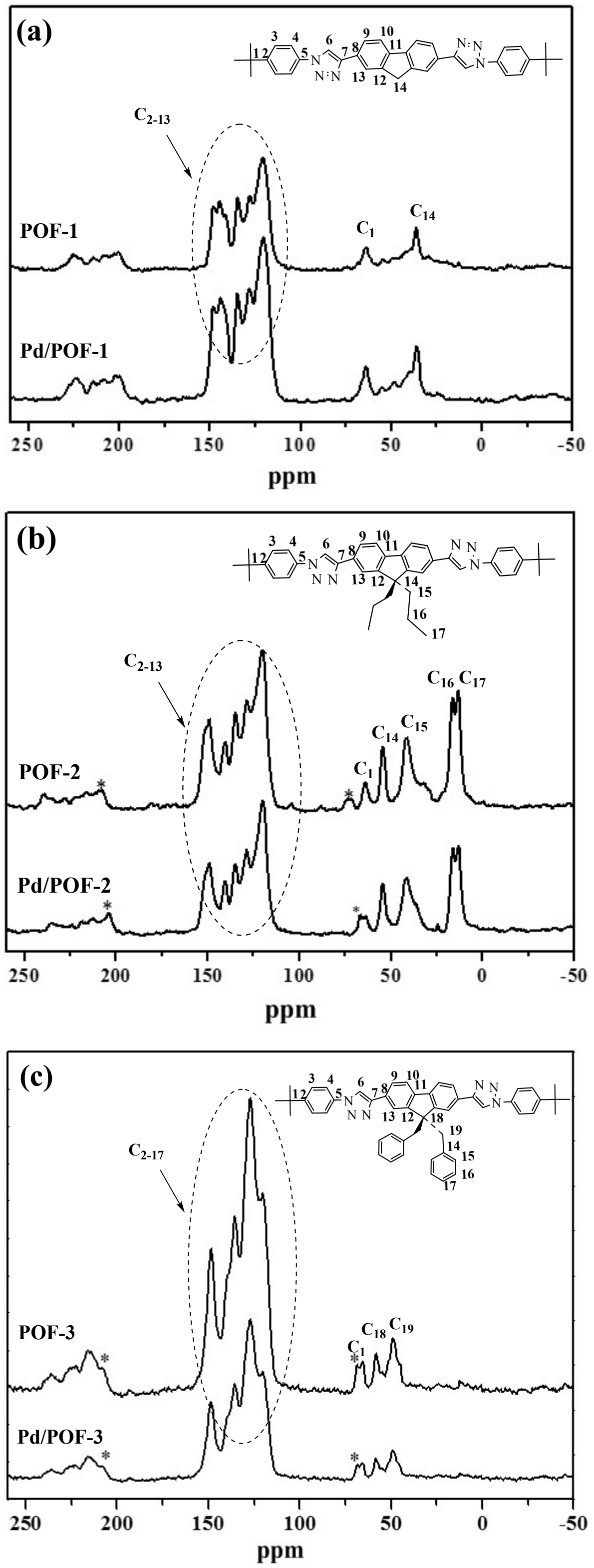

Figure S2. Solid-state ^{13}C NMR spectra for (a) POF-1 and Pd/POF-1; (b) POF-2 and Pd/POF-2; (c) POF-3 and Pd/POF-3.

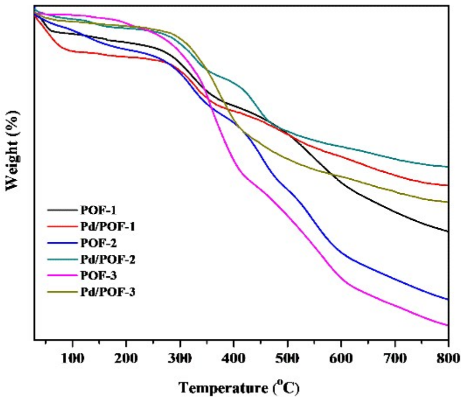

Figure S3. TGA curves for POF-1, POF-2, POF-3, Pd/POF-1, Pd/POF-2 and Pd/POF-3.

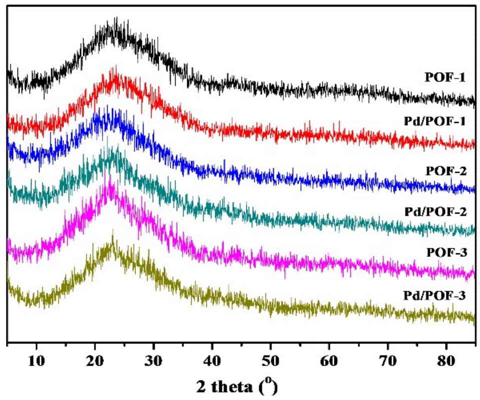

Figure S4. XRD patterns for POF-1, POF-2, POF-3, Pd/POF-1, Pd/POF-2 and Pd/POF-3.

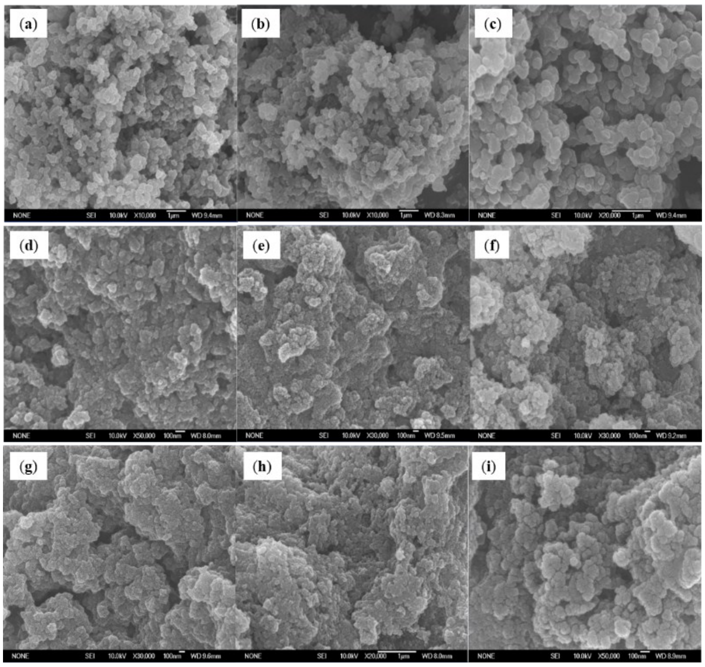

Figure S5. SEM images for POF-1 (a), Pd/POF-1 (b), Pd/POF-1-7run (c), POF-2 (d), Pd/POF-2 (e), Pd/POF-2-6run (f), POF-3 (g), Pd/POF-3 (h) and Pd/POF-3-4run (i).

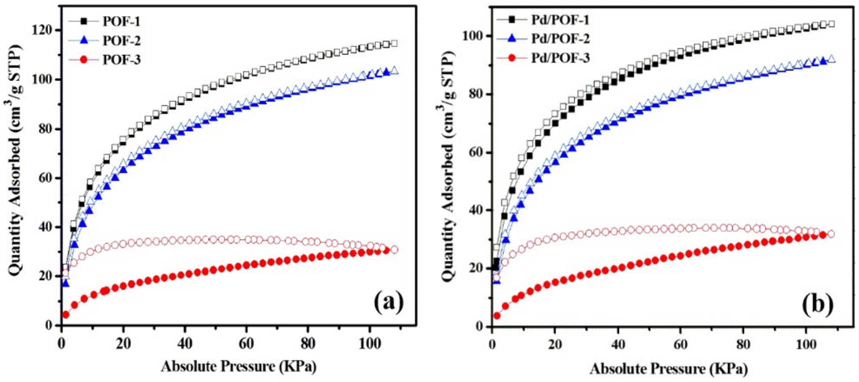

Figure S6. H_2 adsorption/desorption isotherms for (a) POF-1, POF-2 and POF-3; (b) Pd/POF-1, Pd/POF-2 and Pd/POF-3 at 77 K.

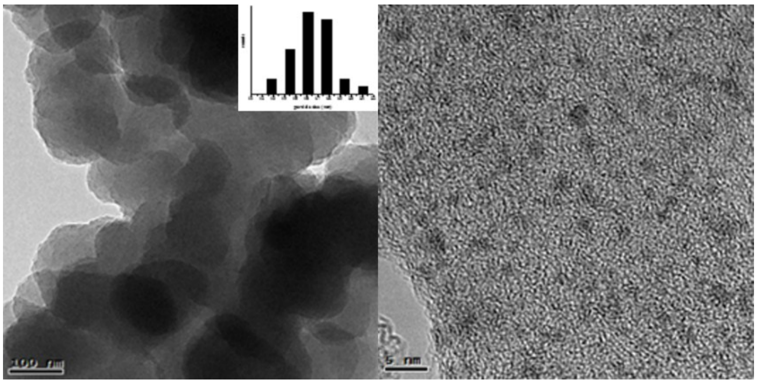

Figure S7. TEM images of Pd/POF-1 prepared with two times of the theoretical amount of $Pd(OAc)_2$

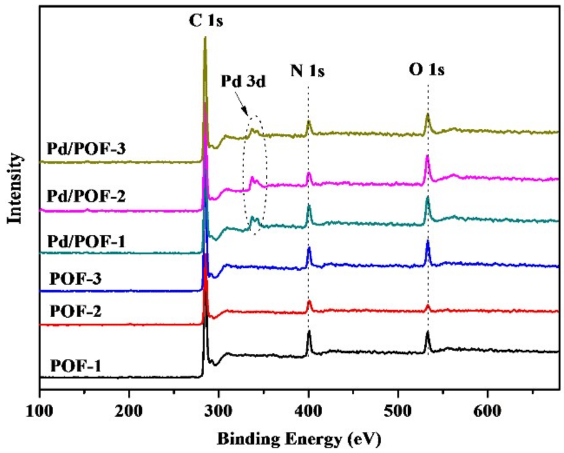

Figure S8. XPS spectra for POF-1, POF-2, POF-3, Pd/POF-1, Pd/POF-2 and Pd/POF-3.

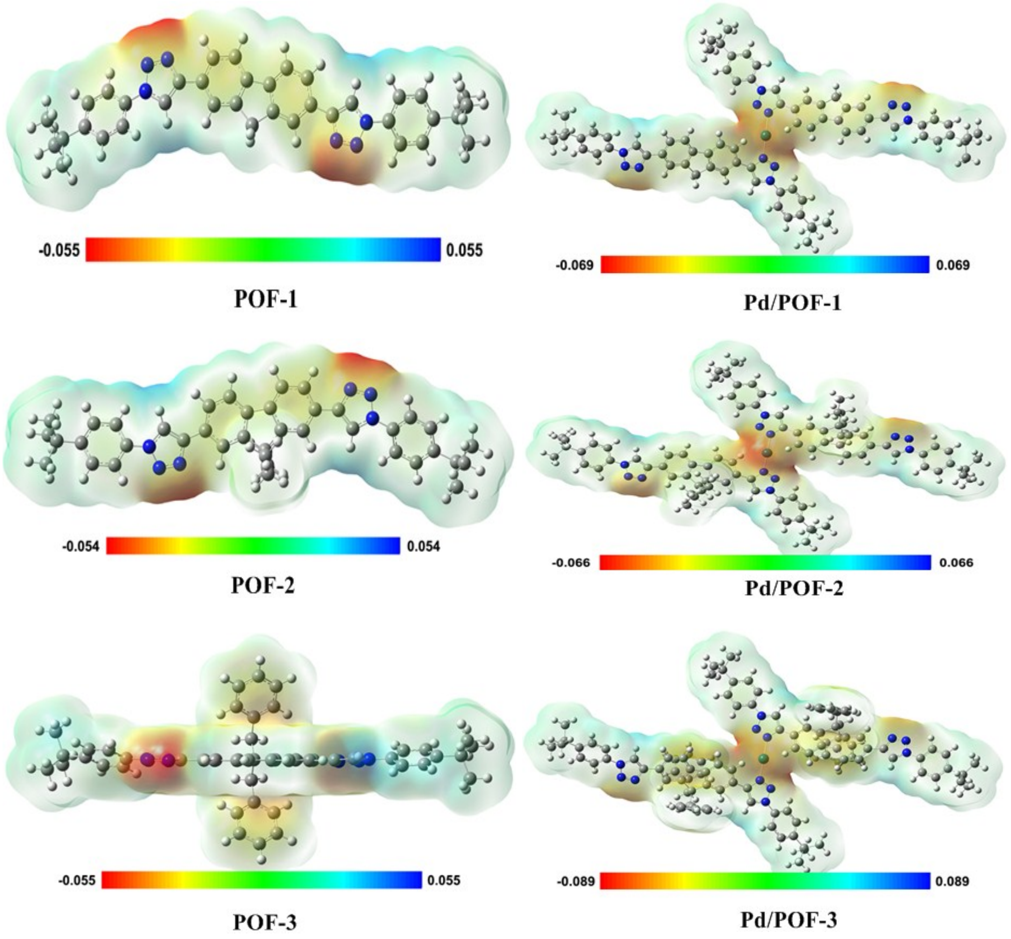

Figure S9. ESP-mapped molecular van der Waals surface for POF-1, POF-2, POF-3, Pd/POF-1, Pd/POF-2 and Pd/POF-3.

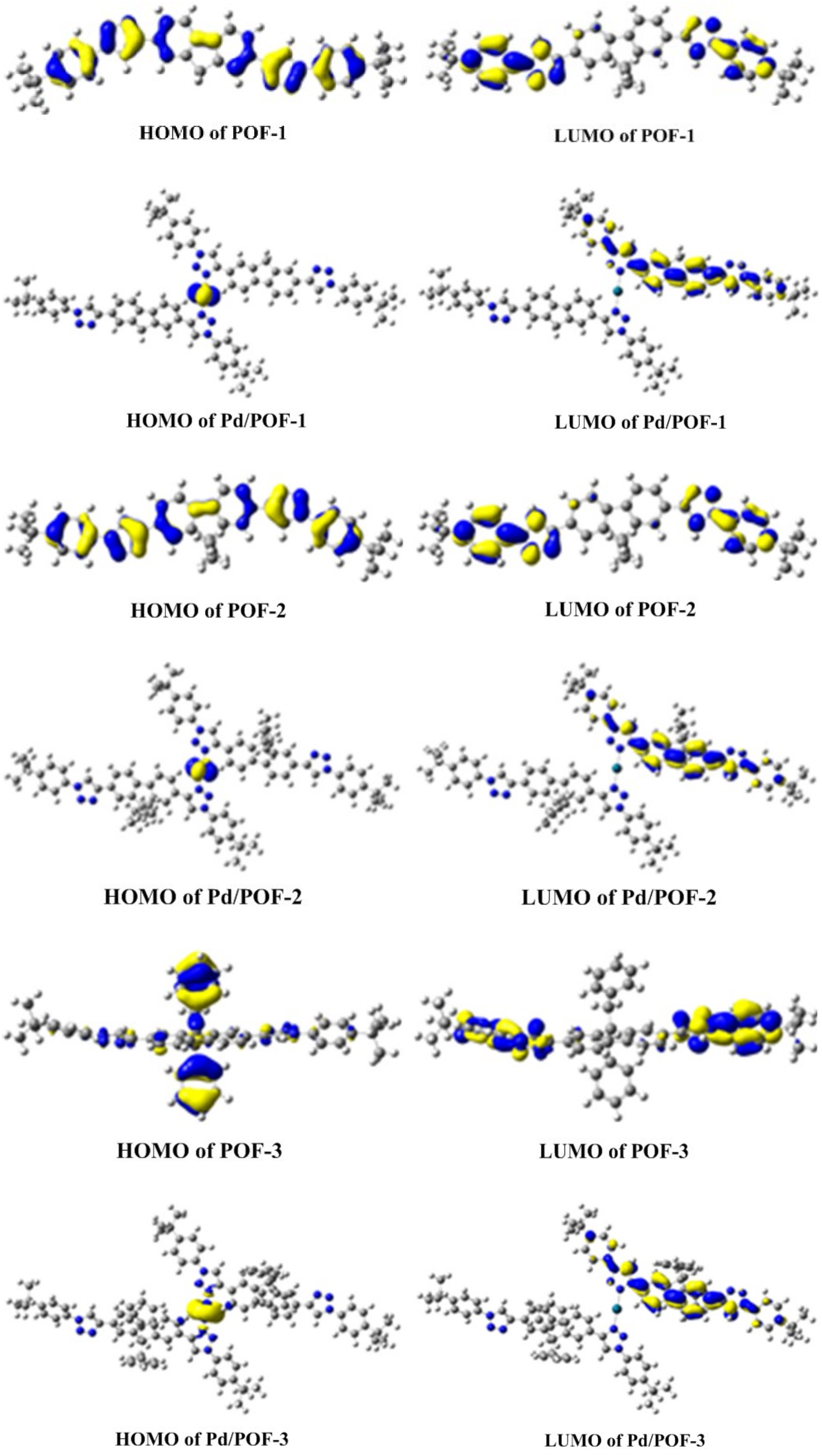

Figure S10. The highest occupied molecular orbital (HOMO) and the lowest unoccupied molecular orbital (LUMO) for POF-1, POF-2, POF-3, Pd/POF-1, Pd/POF-2 and Pd/POF-3.

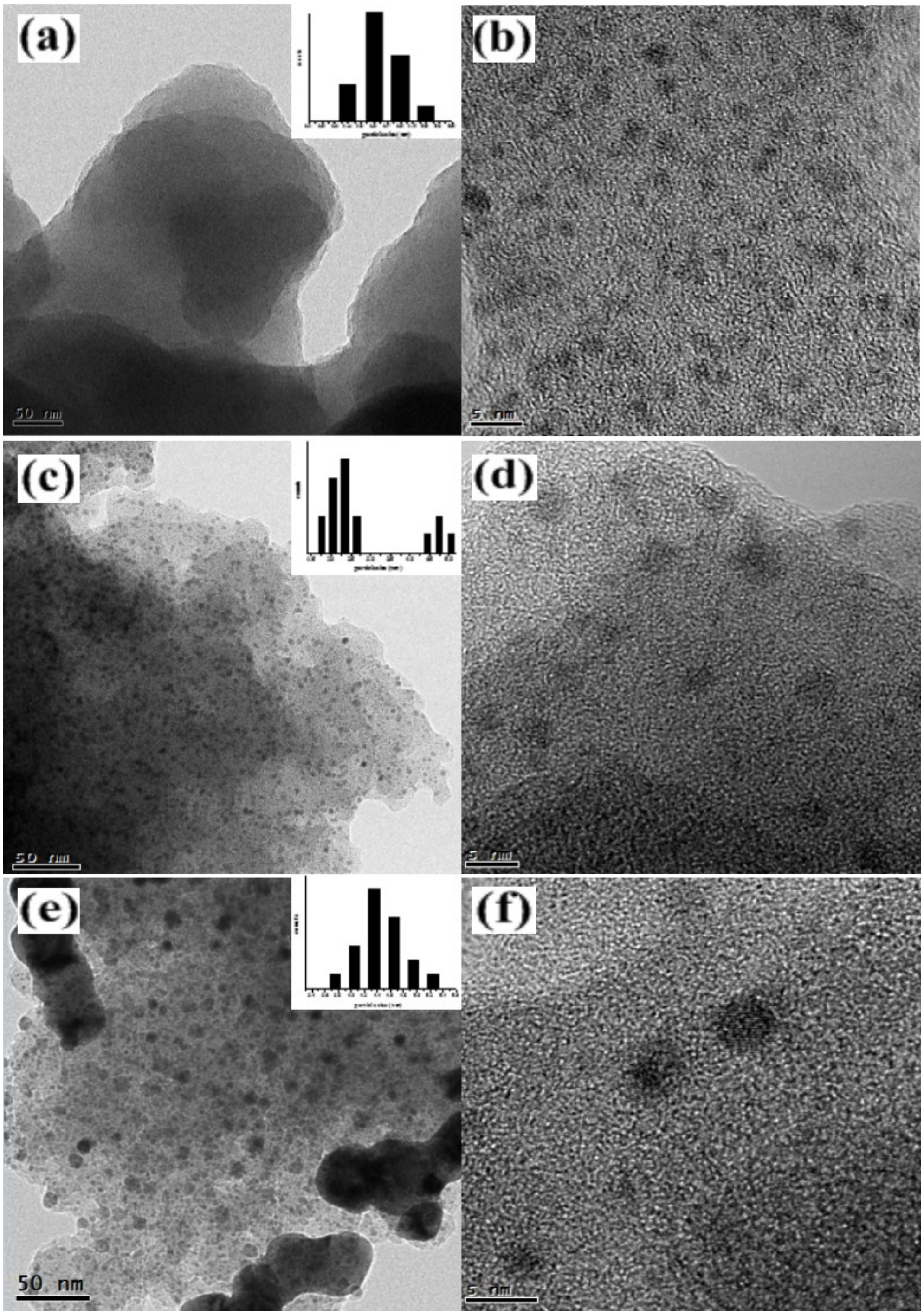

Figure S11. TEM images of Pd/POF-1, Pd/POF-2 and Pd/POF-3 after stored in a specimen tube over half year.

Table S1. Porous properties of POF-1, POF-2, POF-3, Pd/POF-1, Pd/POF-2 and Pd/POF-3.

Polymer	BET ($m^2\ g^{-1}$)	Langmuir ($m^2\ g^{-1}$)	V_{Total} ($cm^3\ g^{-1}$)	pore size distribution (Å)
POF-1	871	1005	0.43	6.7, 12.3, 14.8
POF-2	622	717	0.30	6.4, 8.3, 12.3, 14.8
POF-3	23	65	0.044	-
Pd/POF-1	588	673	0.27	12.1, 14.8, 27.4
Pd/POF-2	438	516	0.20	12.1, 14.8, 27.4
Pd/POF-3	9	19	0.010	-

Table S2. The bond distances, dihedral angles of N2-N1-N4-N5 (β) and angles between pseudo-planes in Pd/POFs

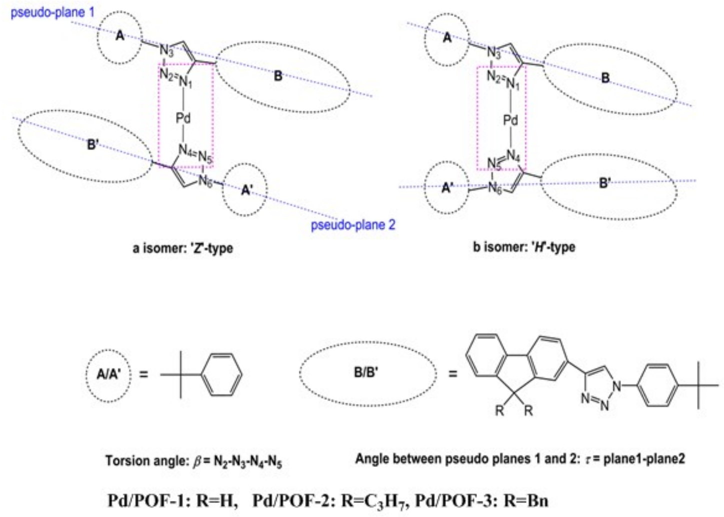

	Pd-N_1 / Å	Pd-N_4 / Å	β / °	τ / °	Relative stability (kcal/mol)
**Pd/POF-1**	2.088	2.088	149.1	28.6	2.13
**Pd/POF-2**	2.084	2.088	153.2	24.6	0.57
**Pd/POF-3**	2.087	2.086	147.9	30.7	2.94

Table S3. Charge distributions of atoms and fragments in POF-1, POF-2, POF-3, Pd/POF-1, Pd/POF-2 and Pd/POF-3.

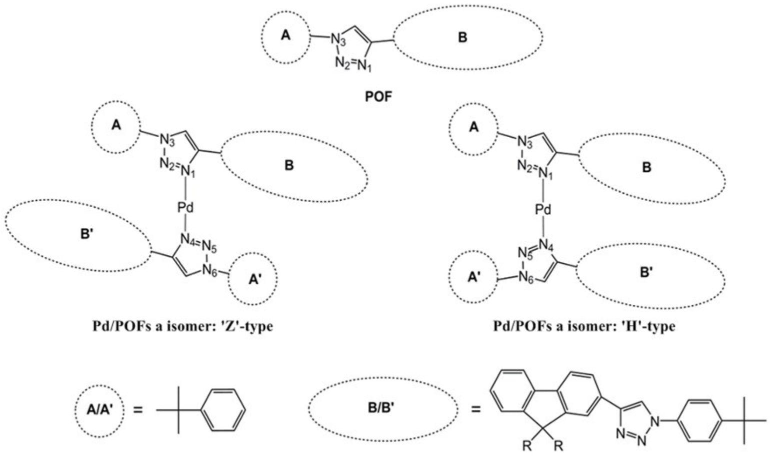

POF-1, Pd/POF-1: R=H; POF-2, Pd/POF-2: R=C_3H_7; POF-3, Pd/POF-3: R=Bn

Atom or fragment	**POF-1**	**Pd/POF-1**	**POF-2**	**Pd/POF-2**	**POF-3**	**Pd/POF-3**
**N1**	-0.24960	-0.28231	-0.24953	-0.28161	-0.24941	-0.28301
**N2**	-0.06102	-0.03936	-0.06099	-0.04191	-0.06041	-0.03832
**N3**	-0.18447	-0.16601	-0.18453	-0.16737	-0.18433	-0.16593
**N4**	–	-0.28219	–	-0.28269	–	-0.28290
**N5**	–	-0.03957	–	-0.03943	–	-0.03832
**N6**	–	-0.16590	–	-0.16660	–	-0.16603
**A**	0.22997	0.22247	0.23001	0.23261	0.23092	0.23360
**B**	0.00373	0.00363	0.00331	0.00705	0.00393	0.00476
**A'**	–	0.23311	–	0.23423	–	0.23424
**B'**	–	0.00264	–	0.00264	–	0.00427

POF-1	atom number	atom type	coordinates (Angstroms)		
			X	Y	Z
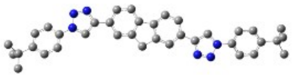	1	C	-12.308241	-1.568187	-0.088995
	2	C	-10.903719	-0.941335	-0.043201
	3	C	-10.696766	0.359190	-0.538066
	4	C	-9.447002	0.966458	-0.524814
	5	C	-8.352921	0.276785	0.007282
	6	C	-8.526621	-1.008473	0.518751
	7	C	-9.787882	-1.605494	0.482123
	8	N	-7.073656	0.895963	0.021719
	9	N	-6.961602	2.253180	0.034147
	10	N	-5.696289	2.540813	0.042937
	11	C	-4.953047	1.385632	0.038288
	12	C	-5.838978	0.323967	0.023679
	13	C	-3.486962	1.404077	0.039114
	14	C	-2.742537	0.209922	0.087931
	15	C	-1.357318	0.261733	0.083791
	16	C	-0.684822	1.501669	0.031952
	17	C	-1.416571	2.691349	-0.014690
	18	C	-2.807296	2.636228	-0.011056
	19	C	0.758486	1.251791	0.041476
	20	C	0.977632	-0.141404	0.101597
	21	C	2.262736	-0.659524	0.124195
	22	C	3.364541	0.216746	0.086606
	23	C	3.136861	1.604525	0.027761
	24	C	1.846671	2.127413	0.004141

25	C	-0.351193	-0.871944	0.132238
26	C	4.725772	-0.327599	0.109077
27	C	5.949613	0.307451	0.006335
28	N	6.871579	-0.690136	0.084272
29	N	6.249918	-1.893115	0.228032
30	N	4.970741	-1.672656	0.241061
31	C	8.289669	-0.607841	0.033212
32	C	9.032803	-1.707253	-0.396478
33	C	10.423658	-1.622324	-0.447030
34	C	11.110427	-0.453070	-0.089558
35	C	10.332404	0.635641	0.339428
36	C	8.944835	0.567311	0.411802
37	C	12.643271	-0.328948	-0.145529
38	H	-9.305890	1.966854	-0.916676
39	H	-7.692236	-1.540754	0.964930
40	H	-9.887926	-2.605720	0.887640
41	H	-5.698781	-0.743621	-0.019737
42	H	-3.252456	-0.749923	0.132665
43	H	-0.913543	3.654192	-0.054030
44	H	-3.393516	3.548128	-0.046895
45	H	2.444401	-1.728774	0.170812
46	H	3.982404	2.287113	0.005299
47	H	1.697041	3.203039	-0.040067
48	H	-0.461883	-1.483047	1.038694
49	H	-0.461033	-1.558128	-0.718930
50	H	6.226720	1.337536	-0.146143
51	H	8.519468	-2.617244	-0.684207

52	H	10.971390	-2.495103	-0.783761
53	H	10.814954	1.561342	0.638831
54	H	8.380233	1.418043	0.780613
55	C	13.186750	-0.021085	1.270369
56	H	12.934220	-0.824851	1.971157
57	H	14.278733	0.077375	1.244679
58	H	12.778757	0.912527	1.672130
59	C	13.315862	-1.620640	-0.647068
60	H	13.103959	-2.472549	0.008873
61	H	12.995473	-1.880686	-1.662377
62	H	14.402841	-1.485090	-0.669510
63	C	13.031726	0.821365	-1.104824
64	H	14.122405	0.927074	-1.149498
65	H	12.667068	0.626226	-2.119697
66	H	12.618281	1.782026	-0.780040
67	C	-13.285581	-0.697622	0.736710
68	H	-14.295181	-1.124838	0.705033
69	H	-12.972190	-0.642634	1.785393
70	H	-13.345038	0.325848	0.352059
71	C	-12.794994	-1.633207	-1.556612
72	H	-12.124541	-2.249887	-2.165952
73	H	-13.798775	-2.072356	-1.604685
74	H	-12.844468	-0.640119	-2.014998
75	C	-12.330601	-2.996176	0.488019
76	H	-11.674744	-3.673471	-0.071048
77	H	-12.027680	-3.016123	1.541110
78	H	-13.347241	-3.400499	0.430552

	79	H	-11.530045	0.917004	-0.955133

Pd/POF-1	center number	atomic type	coordinates (Angstroms)		
			X	Y	Z
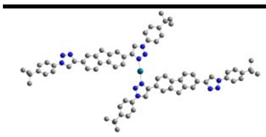	1	C	-4.151553	8.370616	-2.611054
	2	C	-3.126635	7.265337	-2.301728
	3	C	-3.562825	6.004307	-1.855440
	4	C	-2.676940	4.974178	-1.561912
	5	C	-1.303413	5.184610	-1.721697
	6	C	-0.837813	6.418832	-2.173625
	7	C	-1.744720	7.443306	-2.449760
	8	N	-0.390339	4.135717	-1.425600
	9	N	-0.795978	2.847379	-1.466608
	10	N	0.241000	2.107337	-1.152886
	11	C	1.339721	2.908941	-0.905788
	12	C	0.921938	4.213590	-1.079536
	13	C	2.676818	2.436092	-0.526017
	14	C	3.795173	3.255792	-0.781730
	15	C	5.058599	2.838534	-0.393882
	16	C	5.236064	1.593063	0.247182
	17	C	4.133808	0.771195	0.495259
	18	C	2.862752	1.196010	0.113376
	19	C	6.662440	1.415971	0.534214
	20	C	7.363618	2.550112	0.069856
	21	C	8.738078	2.648089	0.219157
	22	C	9.444721	1.601360	0.842697
	23	C	8.738303	0.473470	1.301884

24	C	7.357121	0.372465	1.152197
25	C	6.395979	3.534112	-0.559342
26	C	10.898535	1.705210	1.003762
27	C	11.813654	0.807061	1.521922
28	N	13.018388	1.432542	1.418109
29	N	12.860180	2.664043	0.859731
30	N	11.596104	2.822384	0.613176
31	C	14.307709	0.973101	1.800705
32	C	15.441546	1.496814	1.178612
33	C	16.706208	1.047561	1.557233
34	C	16.882246	0.066636	2.544159
35	C	15.720390	-0.441216	3.150304
36	C	14.449682	0.003746	2.798090
37	C	18.265747	-0.453588	2.972448
38	H	-3.032516	4.010539	-1.216684
39	H	0.223039	6.582761	-2.335101
40	H	-1.349189	8.389633	-2.800097
41	H	1.429754	5.152290	-0.933073
42	H	3.666470	4.202544	-1.301644
43	H	4.253612	-0.192180	0.984239
44	H	1.994598	0.562331	0.288557
45	H	9.288239	3.515376	-0.132393
46	H	9.276281	-0.336124	1.788376
47	H	6.833662	-0.507775	1.516010
48	H	6.634663	3.723476	-1.615191
49	H	6.420414	4.510760	-0.056145
50	H	11.715024	-0.194575	1.906458

51	H	15.325498	2.251193	0.409436
52	H	17.565811	1.476695	1.055492
53	H	15.800131	-1.195274	3.927708
54	H	13.578429	-0.387718	3.313951
55	C	18.347283	-1.975826	2.705969
56	H	18.211727	-2.195712	1.640862
57	H	19.326626	-2.363873	3.011561
58	H	17.582506	-2.529120	3.261540
59	C	19.411698	0.232192	2.204041
60	H	19.345382	0.054332	1.124598
61	H	19.425838	1.314982	2.373308
62	H	20.373391	-0.167119	2.545051
63	C	18.471977	-0.185951	4.482865
64	H	19.453734	-0.554741	4.803934
65	H	18.423802	0.886884	4.701354
66	H	17.713478	-0.686099	5.094268
67	C	-5.114717	7.882759	-3.719943
68	H	-5.857258	8.657847	-3.945356
69	H	-4.568614	7.655925	-4.642593
70	H	-5.656958	6.979031	-3.422626
71	C	-4.962820	8.692430	-1.332754
72	H	-4.307095	9.053188	-0.531991
73	H	-5.706072	9.471661	-1.541004
74	H	-5.497949	7.814123	-0.956794
75	C	-3.481458	9.670838	-3.094419
76	H	-2.806491	10.088563	-2.338476
77	H	-2.910807	9.517627	-4.017607

78	H	-4.248581	10.424774	-3.303489
79	H	-4.624401	5.815619	-1.726083
80	Pd	-0.001333	0.033473	-1.117718
81	C	4.183633	-8.249414	-2.761832
82	C	3.165541	-7.157158	-2.390128
83	C	3.355921	-5.835866	-2.834702
84	C	2.461169	-4.815573	-2.533511
85	C	1.335219	-5.099718	-1.753759
86	C	1.123926	-6.395484	-1.284146
87	C	2.028761	-7.407223	-1.610037
88	N	0.412740	-4.061810	-1.446420
89	N	0.813303	-2.771339	-1.454524
90	N	-0.233450	-2.041517	-1.149602
91	C	-1.331689	-2.852453	-0.933828
92	C	-0.905380	-4.151565	-1.127288
93	C	-2.676812	-2.391196	-0.567623
94	C	-3.787788	-3.214832	-0.841803
95	C	-5.056785	-2.810390	-0.458680
96	C	-5.247385	-1.574585	0.197093
97	C	-4.152571	-0.748032	0.461770
98	C	-2.875999	-1.159118	0.083138
99	C	-6.676174	-1.413649	0.481585
100	C	-7.365770	-2.547927	0.000456
101	C	-8.739273	-2.661687	0.147231
102	C	-9.456776	-1.631219	0.785314
103	C	-8.762100	-0.502553	1.260192
104	C	-7.381895	-0.385682	1.112961

105	C	-6.387741	-3.514242	-0.639918
106	C	-10.909111	-1.753426	0.946743
107	C	-11.833285	-0.873265	1.479474
108	N	-13.030879	-1.511340	1.370053
109	N	-12.859749	-2.733036	0.794251
110	N	-11.594588	-2.873145	0.542343
111	C	-14.324314	-1.072987	1.763470
112	C	-15.454107	-1.603727	1.139975
113	C	-16.722710	-1.175521	1.529662
114	C	-16.906883	-0.209063	2.529287
115	C	-15.748931	0.306483	3.136407
116	C	-14.474225	-0.117707	2.773197
117	C	-18.295043	0.287614	2.970216
118	H	2.621441	-3.805516	-2.891928
119	H	0.272316	-6.621010	-0.649864
120	H	1.833817	-8.403251	-1.229608
121	H	-1.427036	-5.092144	-1.068454
122	H	-3.649702	-4.155677	-1.369913
123	H	-4.282506	0.207743	0.963076
124	H	-2.013200	-0.522076	0.271812
125	H	-9.280381	-3.529717	-0.216307
126	H	-9.308463	0.294489	1.757948
127	H	-6.867470	0.494222	1.490343
128	H	-6.620767	-3.688916	-1.699560
129	H	-6.406143	-4.498798	-0.152193
130	H	-11.745265	0.123928	1.877905
131	H	-15.331869	-2.347192	0.361201

132	H	-17.579054	-1.609735	1.026728
133	H	-15.834789	1.049917	3.923332
134	H	-13.605795	0.278727	3.290014
135	C	-18.397743	1.811718	2.722013
136	H	-18.269312	2.046043	1.659088
137	H	-19.380860	2.183380	3.035739
138	H	-17.638091	2.368437	3.281178
139	C	-19.435559	-0.404090	2.199029
140	H	-19.376414	-0.213143	1.121410
141	H	-19.435064	-1.488829	2.355998
142	H	-20.400726	-0.021048	2.548796
143	C	-18.490459	-0.000440	4.478222
144	H	-19.475566	0.351195	4.808179
145	H	-18.426835	-1.075030	4.683721
146	H	-17.735859	0.502657	5.092010
147	C	4.259229	-8.378190	-4.302275
148	H	4.988115	-9.147830	-4.584020
149	H	3.286710	-8.661250	-4.720974
150	H	4.566500	-7.439854	-4.775770
151	C	5.577058	-7.862607	-2.210779
152	H	5.555203	-7.769931	-1.118982
153	H	6.315432	-8.630142	-2.472780
154	H	5.928459	-6.909593	-2.620006
155	C	3.803363	-9.625316	-2.182398
156	H	3.761833	-9.609994	-1.087211
157	H	2.834421	-9.974218	-2.557910
158	H	4.555303	-10.367456	-2.472854

	159	H	4.222263	-5.590409	-3.441836

POF-2	center number	atomic type	coordinates (Angstroms)		
			X	Y	Z
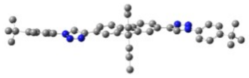	1	C	12.241713	-1.314041	0.086323
	2	C	10.836214	-0.688722	0.049797
	3	C	10.628139	0.605916	0.559429
	4	C	9.377500	1.211523	0.554678
	5	C	8.283633	0.526283	0.016457
	6	C	8.458349	-0.753060	-0.509281
	7	C	9.720485	-1.348674	-0.481053
	8	N	7.003633	1.144072	0.010060
	9	N	6.890147	2.501273	0.010705
	10	N	5.624583	2.787638	0.005748
	11	C	4.882433	1.631658	0.000037
	12	C	5.769548	0.570836	0.003688
	13	C	3.416373	1.648916	0.000131
	14	C	2.672052	0.453699	-0.049948
	15	C	1.285938	0.500503	-0.045565
	16	C	0.616140	1.740766	0.007676
	17	C	1.344948	2.931649	0.055904
	18	C	2.736217	2.880052	0.052265
	19	C	-0.826681	1.490188	-0.002955
	20	C	-1.041043	0.097410	-0.064653
	21	C	-2.327694	-0.418442	-0.090133
	22	C	-3.431574	0.456567	-0.054092

23	C	-3.205498	1.843908	0.007471
24	C	-1.914390	2.365856	0.034218
25	C	0.288552	-0.657238	-0.094916
26	C	-4.791564	-0.090547	-0.082126
27	C	-6.017499	0.540318	0.022171
28	N	-6.936478	-0.459357	-0.064686
29	N	-6.311004	-1.659510	-0.215128
30	N	-5.032484	-1.435423	-0.223562
31	C	-8.355051	-0.382307	-0.017138
32	C	-9.095624	-1.489938	0.395621
33	C	-10.486946	-1.411087	0.441607
34	C	-11.176977	-0.240114	0.096289
35	C	-10.401494	0.857151	-0.315115
36	C	-9.013363	0.795154	-0.382888
37	C	-12.710524	-0.123054	0.147514
38	H	9.235616	2.207374	0.957688
39	H	7.624060	-1.281511	-0.960182
40	H	9.821255	-2.344201	-0.897789
41	H	5.630577	-0.497289	0.036345
42	H	3.185989	-0.504109	-0.095714
43	H	0.839716	3.893386	0.096522
44	H	3.320861	3.792887	0.089443
45	H	-2.512276	-1.487211	-0.138882
46	H	-4.051060	2.526533	0.028810
47	H	-1.764399	3.441459	0.079815
48	H	-6.297587	1.568644	0.180909
49	H	-8.579967	-2.401646	0.673500

50	H	-11.032444	-2.290384	0.764695
51	H	-10.886493	1.784800	-0.604316
52	H	-8.450893	1.652926	-0.738377
53	C	-13.249421	0.207346	-1.265035
54	H	-12.990567	-0.582875	-1.978789
55	H	-14.341898	0.300643	-1.242090
56	H	-12.843989	1.149654	-1.648719
57	C	-13.379854	-1.426239	0.623092
58	H	-13.160352	-2.265598	-0.046400
59	H	-13.063854	-1.702531	1.635473
60	H	-14.467553	-1.296020	0.641896
61	C	-13.107735	1.008524	1.125315
62	H	-14.199032	1.108968	1.167408
63	H	-12.746368	0.796971	2.138095
64	H	-12.696890	1.976434	0.819257
65	C	13.216964	-0.431728	-0.729322
66	H	14.227030	-0.858298	-0.704708
67	H	12.901954	-0.363321	-1.776726
68	H	13.276011	0.586643	-0.331335
69	C	12.730096	-1.396826	1.552463
70	H	12.060922	-2.021624	2.154900
71	H	13.734347	-1.835601	1.593936
72	H	12.779104	-0.409390	2.022938
73	C	12.265571	-2.734554	-0.508844
74	H	11.611559	-3.419995	0.042396
75	H	11.961295	-2.741503	-1.561696
76	H	13.282963	-3.137896	-0.457804

	77	H	11.461230	1.160357	0.981346
	78	C	0.442430	-1.507197	-1.390773
	79	C	0.325834	-0.752794	-2.721335
	80	H	-0.314506	-2.303682	-1.362031
	81	H	1.417202	-2.015018	-1.349331
	82	C	0.475099	-1.683504	-3.930108
	83	H	-0.645009	-0.244681	-2.770958
	84	H	1.089876	0.033029	-2.768248
	85	H	0.391628	-1.127702	-4.870951
	86	H	1.449465	-2.188413	-3.927996
	87	H	-0.299586	-2.460238	-3.932811
	88	C	0.441059	-1.611380	1.126655
	89	H	1.417703	-2.110701	1.046096
	90	H	-0.312983	-2.405264	1.029884
	91	C	0.318568	-0.970321	2.514783
	92	H	-0.656253	-0.475702	2.605370
	93	H	1.076291	-0.184881	2.626360
	94	C	0.475631	-1.996506	3.642569
	95	H	1.454432	-2.490783	3.599519
	96	H	0.386814	-1.521458	4.626191
	97	H	-0.292023	-2.777735	3.579843

**Pd/POF-2**	center number	atomic type	coordinates (Angstroms)		
			X	Y	Z
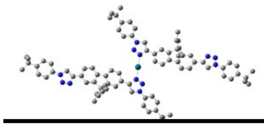	1	C	-3.722876	8.845094	-2.106610
	2	C	-2.767040	7.662149	-1.872799

3	C	-3.271512	6.424170	-1.433843
4	C	-2.448316	5.328006	-1.203463
5	C	-1.072158	5.445974	-1.422714
6	C	-0.540605	6.654779	-1.870406
7	C	-1.383982	7.746714	-2.082118
8	N	-0.222956	4.330587	-1.187660
9	N	-0.705938	3.071779	-1.284862
10	N	0.285466	2.257911	-1.007464
11	C	1.429214	2.980793	-0.726544
12	C	1.092324	4.314275	-0.843105
13	C	2.730620	2.408411	-0.361094
14	C	3.908314	3.123373	-0.661500
15	C	5.142473	2.613026	-0.286798
16	C	5.222573	1.378513	0.391298
17	C	4.061827	0.659846	0.686903
18	C	2.822482	1.176708	0.313130
19	C	6.634038	1.087350	0.656345
20	C	7.416656	2.139267	0.135487
21	C	8.798222	2.116196	0.252479
22	C	9.426728	1.032165	0.897499
23	C	8.636205	-0.012418	1.411540
24	C	7.247737	0.007401	1.296088
25	C	6.533726	3.203234	-0.517487
26	C	10.887780	1.011614	1.020495
27	C	11.726126	0.109058	1.649292
28	N	12.981782	0.594030	1.445660
29	N	12.927258	1.746794	0.723500

30	N	11.678094	1.993054	0.473260
31	C	14.231249	0.067238	1.871426
32	C	15.327066	0.918647	2.017127
33	C	16.553788	0.400485	2.431007
34	C	16.725575	-0.960642	2.721988
35	C	15.601941	-1.790821	2.568030
36	C	14.372670	-1.297174	2.141708
37	C	18.065843	-1.558601	3.185618
38	H	-2.854139	4.384472	-0.857731
39	H	0.521206	6.745912	-2.077396
40	H	-0.938785	8.671376	-2.430839
41	H	1.655248	5.213362	-0.654121
42	H	3.842676	4.060966	-1.209466
43	H	4.111771	-0.294124	1.205573
44	H	1.909311	0.624879	0.524729
45	H	9.416887	2.916112	-0.142640
46	H	9.112720	-0.855455	1.905273
47	H	6.657275	-0.811030	1.699687
48	H	11.537771	-0.779374	2.229098
49	H	15.212282	1.974469	1.802160
50	H	17.384848	1.088960	2.531321
51	H	15.683079	-2.854505	2.771672
52	H	13.537454	-1.976210	2.001178
53	C	18.523445	-2.638102	2.175250
54	H	18.664756	-2.206894	1.177686
55	H	19.476407	-3.077481	2.494251
56	H	17.795430	-3.451588	2.087669

57	C	19.176936	-0.496263	3.287728
58	H	19.375395	-0.018692	2.321490
59	H	18.929364	0.286760	4.013519
60	H	20.108142	-0.969425	3.618946
61	C	17.885196	-2.205378	4.579865
62	H	18.830229	-2.645518	4.920603
63	H	17.570325	-1.461054	5.320194
64	H	17.133185	-3.001487	4.565764
65	C	-4.763941	8.458887	-3.184626
66	H	-5.455080	9.292020	-3.361638
67	H	-4.273944	8.216161	-4.134378
68	H	-5.360082	7.590343	-2.885293
69	C	-4.453890	9.184537	-0.785284
70	H	-3.739940	9.466828	-0.003154
71	H	-5.142340	10.024795	-0.936568
72	H	-5.039571	8.337404	-0.413025
73	C	-2.982853	10.108751	-2.584840
74	H	-2.245470	10.453532	-1.850704
75	H	-2.467567	9.944019	-3.538093
76	H	-3.701773	10.921688	-2.735663
77	H	-4.336604	6.308015	-1.256486
78	Pd	-0.036685	0.200829	-1.096548
79	C	3.960506	-8.135760	-2.902387
80	C	2.957835	-7.026746	-2.537802
81	C	3.217194	-5.694137	-2.907683
82	C	2.339833	-4.657941	-2.609996
83	C	1.161229	-4.938031	-1.911146

84	C	0.879178	-6.245576	-1.517815
85	C	1.768579	-7.272704	-1.838637
86	N	0.256395	-3.883934	-1.608342
87	N	0.705295	-2.615213	-1.488802
88	N	-0.337657	-1.860659	-1.234002
89	C	-1.481527	-2.634704	-1.176453
90	C	-1.089293	-3.935753	-1.423494
91	C	-2.837959	-2.140100	-0.908943
92	C	-3.801526	-3.024537	-0.379526
93	C	-5.096796	-2.584405	-0.153018
94	C	-5.452660	-1.250121	-0.441350
95	C	-4.503980	-0.365055	-0.958027
96	C	-3.204986	-0.811996	-1.194159
97	C	-6.864980	-1.063863	-0.099268
98	C	-7.372485	-2.281663	0.400779
99	C	-8.696218	-2.382953	0.801839
100	C	-9.541698	-1.259093	0.710617
101	C	-9.024039	-0.049473	0.211397
102	C	-7.695215	0.055829	-0.193589
103	C	-6.288117	-3.360292	0.410072
104	C	-10.940960	-1.367276	1.135474
105	C	-11.960573	-0.433119	1.116688
106	N	-13.043153	-1.083225	1.624741
107	N	-12.712862	-2.364459	1.944804
108	N	-11.459638	-2.530306	1.650835
109	C	-14.363227	-0.600834	1.836001
110	C	-15.432806	-1.496458	1.861195

111	C	-16.727093	-1.019947	2.068115
112	C	-16.997463	0.345324	2.241825
113	C	-15.898461	1.220777	2.211141
114	C	-14.597593	0.764968	2.020738
115	C	-18.416479	0.898651	2.461932
116	H	2.553577	-3.638523	-2.909486
117	H	-0.016174	-6.467174	-0.945153
118	H	1.519974	-8.277733	-1.517725
119	H	-1.655886	-4.845386	-1.534514
120	H	-3.517775	-4.044325	-0.128522
121	H	-4.765140	0.665890	-1.183342
122	H	-2.451429	-0.132219	-1.589573
123	H	-9.105178	-3.311301	1.188636
124	H	-9.668248	0.823136	0.140385
125	H	-7.316716	1.000342	-0.575729
126	H	-12.011056	0.584993	0.767666
127	H	-15.243886	-2.554413	1.722049
128	H	-17.535612	-1.741833	2.083392
129	H	-16.051476	2.286817	2.350857
130	H	-13.770732	1.468174	2.037333
131	C	-18.760216	1.898249	1.331610
132	H	-18.730019	1.408189	0.351766
133	H	-19.768236	2.305153	1.477446
134	H	-18.062333	2.741986	1.305011
135	C	-19.483645	-0.212383	2.460577
136	H	-19.511320	-0.751558	1.506694
137	H	-19.316022	-0.941219	3.261779

138	H	-20.474238	0.228504	2.618286
139	C	-18.479571	1.626265	3.826425
140	H	-19.485115	2.030398	3.995357
141	H	-18.246297	0.939824	4.648255
142	H	-17.773055	2.461628	3.878094
143	C	4.096813	-8.216405	-4.442030
144	H	4.819842	-8.993058	-4.719707
145	H	3.136262	-8.463837	-4.908273
146	H	4.442689	-7.270222	-4.871325
147	C	5.339907	-7.808000	-2.281825
148	H	5.275049	-7.749799	-1.189301
149	H	6.066674	-8.588266	-2.538618
150	H	5.735991	-6.853491	-2.643830
151	C	3.518216	-9.517780	-2.385261
152	H	3.425358	-9.534175	-1.293263
153	H	2.559687	-9.828009	-2.816865
154	H	4.263613	-10.270824	-2.664223
155	H	4.125428	-5.451801	-3.451374
156	C	6.851982	3.368097	-2.033292
157	C	6.747014	2.102394	-2.894089
158	H	7.867267	3.780372	-2.121939
159	H	6.176715	4.135600	-2.438971
160	C	7.085196	2.372063	-4.364859
161	H	7.423069	1.334028	-2.499269
162	H	5.732899	1.690522	-2.821691
163	H	7.003317	1.457907	-4.963882
164	H	6.406254	3.115964	-4.800589

165	H	8.108328	2.752591	-4.475818
166	C	6.700972	4.593315	0.163412
167	C	6.426754	4.651329	1.672017
168	H	6.036931	5.304080	-0.350165
169	H	7.724779	4.943814	-0.029629
170	C	6.628316	6.058976	2.245167
171	H	5.401128	4.317443	1.872753
172	H	7.088512	3.947965	2.192148
173	H	6.424843	6.081947	3.321943
174	H	7.657572	6.407216	2.093604
175	H	5.960202	6.785476	1.765102
176	C	-6.671520	-4.572431	-0.489836
177	C	-6.987451	-4.259481	-1.958236
178	H	-7.539172	-5.070657	-0.034345
179	H	-5.847580	-5.299899	-0.446113
180	C	-7.354917	-5.517384	-2.753734
181	H	-7.814521	-3.540888	-2.009139
182	H	-6.123307	-3.770326	-2.424616
183	H	-7.575667	-5.275104	-3.799619
184	H	-6.535405	-6.247389	-2.747486
185	H	-8.239662	-6.010863	-2.332509
186	C	-6.011947	-3.895035	1.846111
187	C	-5.612881	-2.852475	2.898369
188	H	-5.220878	-4.656256	1.777077
189	H	-6.912297	-4.424951	2.188458
190	C	-5.361216	-3.483609	4.272615
191	H	-4.710720	-2.322648	2.568668

	192	H	-6.403225	-2.096533	2.983613
	193	H	-5.077745	-2.724909	5.010980
	194	H	-6.257117	-3.994526	4.647194
	195	H	-4.551928	-4.223591	4.229603

**POF-3**	center number	atomic type	coordinates (Angstroms)		
			X	Y	Z
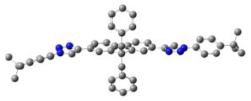	1	C	-12.248141	0.183125	-1.381165
	2	C	-10.844174	0.108282	-0.756127
	3	C	-10.632518	0.569846	0.555819
	4	C	-9.382939	0.529805	1.162305
	5	C	-8.294217	0.004018	0.459545
	6	C	-8.472599	-0.474510	-0.837652
	7	C	-9.733528	-0.411552	-1.433224
	8	N	-7.015059	-0.037842	1.077948
	9	N	-6.903097	-0.096940	2.433736
	10	N	-5.637803	-0.121335	2.720816
	11	C	-4.894352	-0.080640	1.566458
	12	C	-5.780406	-0.026129	0.505931
	13	C	-3.428176	-0.087832	1.585670
	14	C	-2.681923	-0.076148	0.391155
	15	C	-1.295458	-0.077964	0.439839
	16	C	-0.627352	-0.093528	1.680163
	17	C	-1.357731	-0.107352	2.871005
	18	C	-2.748478	-0.103848	2.818231
	19	C	0.814665	-0.092557	1.431638

20	C	1.032477	-0.076632	0.039369
21	C	2.320437	-0.073787	-0.473238
22	C	3.421686	-0.087415	0.404776
23	C	3.191743	-0.104439	1.792523
24	C	1.899550	-0.105978	2.311036
25	C	-0.295654	-0.064466	-0.714158
26	C	4.782508	-0.084075	-0.140593
27	C	6.007122	-0.004557	0.496238
28	N	6.927563	-0.038150	-0.505396
29	N	6.304750	-0.133203	-1.712156
30	N	5.025620	-0.159047	-1.490388
31	C	8.345755	0.014629	-0.423032
32	C	9.085178	0.499012	-1.501965
33	C	10.475936	0.551805	-1.416547
34	C	11.165899	0.142561	-0.266319
35	C	10.391499	-0.341145	0.801917
36	C	9.004153	-0.416259	0.732100
37	C	12.698688	0.200151	-0.141454
38	H	-9.237940	0.896039	2.171853
39	H	-7.642392	-0.915675	-1.380601
40	H	-9.837713	-0.791858	-2.442880
41	H	-5.641230	0.051587	-0.559903
42	H	-3.196178	-0.067575	-0.568033
43	H	-0.853355	-0.120150	3.833751
44	H	-3.333782	-0.113501	3.731329
45	H	2.510104	-0.061893	-1.542350
46	H	4.035476	-0.123026	2.477454

47	H	1.745934	-0.120593	3.386809
48	H	6.285185	0.106460	1.531360
49	H	8.569236	0.827280	-2.396650
50	H	11.020986	0.932224	-2.272878
51	H	10.876692	-0.681730	1.711911
52	H	8.442264	-0.827236	1.565127
53	C	13.249303	-1.226540	0.095538
54	H	12.999695	-1.887790	-0.741934
55	H	14.341208	-1.200642	0.194899
56	H	12.843392	-1.675227	1.008295
57	C	13.367265	0.768989	-1.407075
58	H	13.158200	0.155435	-2.290691
59	H	13.041163	1.794318	-1.615600
60	H	14.454224	0.790418	-1.271249
61	C	13.083795	1.102683	1.055093
62	H	14.174377	1.147544	1.161412
63	H	12.713690	2.124181	0.911830
64	H	12.673655	0.727667	1.998757
65	C	-13.234386	-0.651472	-0.529301
66	H	-14.243592	-0.598571	-0.955320
67	H	-12.932872	-1.704626	-0.498730
68	H	-13.289530	-0.290050	0.502846
69	C	-12.718698	1.657138	-1.412770
70	H	-12.042477	2.272389	-2.017163
71	H	-13.722607	1.726308	-1.848723
72	H	-12.761403	2.094235	-0.409737
73	C	-12.277008	-0.361085	-2.821889

74	H	-11.615854	0.206496	-3.486827
75	H	-11.984923	-1.416542	-2.866134
76	H	-13.293138	-0.284185	-3.224133
77	H	-11.461763	0.981859	1.123287
78	C	-0.455059	-1.310665	-1.652224
79	H	0.271092	-1.200616	-2.467056
80	H	-1.448450	-1.247232	-2.115342
81	C	-0.451773	1.209128	-1.615911
82	H	-1.438201	1.153049	-2.094457
83	H	0.286342	1.129342	-2.423534
84	C	-0.280278	-2.675830	-1.017049
85	C	0.962271	-3.324547	-1.051648
86	C	-1.354737	-3.336504	-0.404452
87	C	1.131032	-4.586792	-0.481608
88	H	1.806344	-2.836657	-1.531981
89	C	-1.190468	-4.598157	0.168274
90	H	-2.329872	-2.858292	-0.373858
91	C	0.054620	-5.227847	0.133204
92	H	2.103783	-5.070171	-0.522848
93	H	-2.038179	-5.090366	0.638140
94	H	0.183007	-6.212361	0.575572
95	C	-0.297804	2.555097	-0.936009
96	C	0.945255	3.203079	-0.906727
97	C	-1.393372	3.199550	-0.344023
98	C	1.093711	4.447954	-0.294435
99	H	1.805795	2.727544	-1.369715
100	C	-1.249795	4.443880	0.270574

	101	H	-2.369160	2.722014	-0.362990
	102	C	-0.004041	5.072479	0.299383
	103	H	2.067456	4.931070	-0.286295
	104	H	-2.113930	4.923468	0.723242
	105	H	0.108549	6.043474	0.774675

**Pd/POF-3**	center number	atomic type	coordinates (Angstroms)		
			X	Y	Z
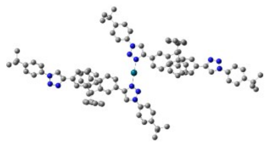	1	C	-3.726993	8.507007	-2.526742
	2	C	-2.758724	7.352460	-2.215170
	3	C	-3.257209	6.117958	-1.759963
	4	C	-2.424015	5.046087	-1.461978
	5	C	-1.042023	5.185770	-1.626274
	6	C	-0.515452	6.391366	-2.088398
	7	C	-1.369996	7.458905	-2.368962
	8	N	-0.183343	4.093664	-1.322813
	9	N	-0.656161	2.828187	-1.351208
	10	N	0.339671	2.038082	-1.027238
	11	C	1.478448	2.783068	-0.785215
	12	C	1.131025	4.105991	-0.974517
	13	C	2.785347	2.238527	-0.396597
	14	C	3.955230	2.964749	-0.700706
	15	C	5.194771	2.476593	-0.313487
	16	C	5.289956	1.253441	0.380010
	17	C	4.137837	0.523439	0.680502
	18	C	2.893297	1.017774	0.295334
	19	C	6.703167	0.984023	0.651944

20	C	7.474282	2.037562	0.120905
21	C	8.855704	2.030341	0.239845
22	C	9.494922	0.960878	0.897232
23	C	8.714838	-0.085576	1.422782
24	C	7.327008	-0.081498	1.305336
25	C	6.578450	3.079107	-0.545378
26	C	10.956090	0.956818	1.018862
27	C	11.805243	0.066175	1.649933
28	N	13.055299	0.563397	1.440991
29	N	12.987400	1.711875	0.713841
30	N	11.734792	1.943891	0.465221
31	C	14.310990	0.050105	1.865401
32	C	15.399115	0.912176	2.005599
33	C	16.631604	0.406768	2.418244
34	C	16.816620	-0.951765	2.713291
35	C	15.700428	-1.792846	2.564655
36	C	14.465646	-1.312059	2.139709
37	C	18.163376	-1.535912	3.175713
38	H	-2.827261	4.104149	-1.109307
39	H	0.551917	6.500082	-2.253814
40	H	-0.928019	8.381531	-2.727144
41	H	1.686960	5.018470	-0.836785
42	H	3.879245	3.893581	-1.262844
43	H	4.198962	-0.422992	1.211361
44	H	1.986720	0.454339	0.508829
45	H	9.468369	2.830941	-0.163697
46	H	9.199614	-0.917639	1.926894

47	H	6.744260	-0.901148	1.717109
48	H	11.627422	-0.821799	2.233707
49	H	15.274096	1.966191	1.787423
50	H	17.456577	1.103149	2.514247
51	H	15.792012	-2.855045	2.771449
52	H	13.636465	-1.999271	2.003311
53	C	18.628495	-2.614370	2.167646
54	H	18.763294	-2.185147	1.168330
55	H	19.586265	-3.043884	2.485662
56	H	17.907863	-3.434854	2.084550
57	C	19.264824	-0.463046	3.271967
58	H	19.456626	0.013396	2.303828
59	H	19.011676	0.319852	3.995961
60	H	20.201134	-0.926572	3.602418
61	C	17.991802	-2.180013	4.572383
62	H	18.941726	-2.610058	4.912362
63	H	17.671487	-1.436402	5.311086
64	H	17.247390	-2.983320	4.562515
65	C	-4.712307	8.065780	-3.635623
66	H	-5.413044	8.877547	-3.865933
67	H	-4.177038	7.807053	-4.556254
68	H	-5.301685	7.192799	-3.335970
69	C	-4.522086	8.870845	-1.249605
70	H	-3.849717	9.197901	-0.448242
71	H	-5.224063	9.687046	-1.459239
72	H	-5.102076	8.021439	-0.873839
73	C	-2.992645	9.771331	-3.011756

74	H	-2.294774	10.153742	-2.257866
75	H	-2.433512	9.589500	-3.936757
76	H	-3.720854	10.563601	-3.218004
77	H	-4.326487	5.983946	-1.625905
78	Pd	0.002158	-0.020664	-0.969732
79	C	3.686382	-8.548109	-2.600954
80	C	2.737442	-7.396502	-2.225468
81	C	2.994130	-6.093362	-2.689814
82	C	2.164916	-5.020456	-2.384424
83	C	1.039493	-5.231383	-1.581104
84	C	0.762847	-6.507818	-1.092869
85	C	1.602753	-7.573063	-1.422655
86	N	0.185436	-4.137770	-1.268311
87	N	0.663620	-2.874201	-1.292401
88	N	-0.331771	-2.080119	-0.977296
89	C	-1.473464	-2.820934	-0.737763
90	C	-1.131001	-4.144861	-0.928338
91	C	-2.779462	-2.269452	-0.355335
92	C	-3.953171	-2.985616	-0.667260
93	C	-5.190122	-2.492875	-0.277429
94	C	-5.278923	-1.276035	0.427634
95	C	-4.122755	-0.554848	0.733984
96	C	-2.880848	-1.052499	0.344363
97	C	-6.690494	-1.004234	0.705874
98	C	-7.466793	-2.049860	0.166585
99	C	-8.847336	-2.042143	0.294957
100	C	-9.480267	-0.981507	0.972321

101	C	-8.695159	0.056846	1.506295
102	C	-7.308395	0.053866	1.376671
103	C	-6.576839	-3.084896	-0.517402
104	C	-10.939885	-0.980496	1.110878
105	C	-11.793277	-0.030546	1.641636
106	N	-13.039556	-0.560918	1.504138
107	N	-12.965555	-1.784843	0.913515
108	N	-11.712763	-2.031033	0.679366
109	C	-14.296335	-0.014124	1.880855
110	C	-15.456579	-0.429987	1.226873
111	C	-16.688623	0.107668	1.598183
112	C	-16.804099	1.073064	2.609097
113	C	-15.616787	1.470558	3.247720
114	C	-14.379110	0.936034	2.903052
115	C	-18.148757	1.692035	3.029841
116	H	2.376595	-4.024935	-2.756515
117	H	-0.088367	-6.677383	-0.440802
118	H	1.359331	-8.552186	-1.026553
119	H	-1.707711	-5.051752	-0.854020
120	H	-3.882771	-3.910909	-1.235885
121	H	-4.178919	0.386456	1.274546
122	H	-1.971187	-0.496306	0.562336
123	H	-9.463745	-2.837480	-0.113234
124	H	-9.174420	0.875499	2.036955
125	H	-6.721383	0.865190	1.798886
126	H	-11.625917	0.951112	2.052939
127	H	-15.386578	-1.169957	0.438317

128	H	-17.570336	-0.238188	1.070933
129	H	-15.650974	2.207619	4.044607
130	H	-13.488942	1.243403	3.443013
131	C	-18.103614	3.221726	2.799612
132	H	-17.938496	3.454554	1.741431
133	H	-19.051809	3.680533	3.105258
134	H	-17.302980	3.697665	3.375764
135	C	-19.332771	1.119635	2.227425
136	H	-19.233701	1.317835	1.154068
137	H	-19.437067	0.037811	2.368807
138	H	-20.264926	1.587268	2.563682
139	C	-18.401187	1.406843	4.529975
140	H	-19.355785	1.845751	4.844558
141	H	-18.442295	0.328842	4.722961
142	H	-17.616012	1.830814	5.164855
143	C	3.721421	-8.702719	-4.140504
144	H	4.401069	-9.514930	-4.425671
145	H	2.726104	-8.938058	-4.534447
146	H	4.068783	-7.789327	-4.634639
147	C	5.110146	-8.229152	-2.084985
148	H	5.116673	-8.120136	-0.994495
149	H	5.800782	-9.038775	-2.350533
150	H	5.503207	-7.302198	-2.515518
151	C	3.245084	-9.892743	-1.992283
152	H	3.228582	-9.859624	-0.896818
153	H	2.250757	-10.193643	-2.341913
154	H	3.949173	-10.678830	-2.287014

155	H	3.861617	-5.904761	-3.315380
156	C	6.901697	3.246220	-2.070891
157	H	7.890767	3.715196	-2.146223
158	H	6.185554	3.970824	-2.479921
159	C	6.727338	4.494612	0.111598
160	H	6.023518	5.167124	-0.396099
161	H	7.732026	4.863341	-0.129526
162	C	-6.899872	-3.221358	-2.046360
163	H	-7.890715	-3.684868	-2.131368
164	H	-6.186126	-3.940441	-2.469092
165	C	-6.732769	-4.510782	0.113895
166	H	-6.026444	-5.175886	-0.400075
167	H	-7.736102	-4.874192	-0.140477
168	C	6.885590	1.987996	-2.916557
169	C	8.068927	1.274842	-3.156946
170	C	5.698850	1.517401	-3.497161
171	C	8.066421	0.121144	-3.942098
172	H	9.001970	1.627302	-2.725197
173	C	5.690864	0.362981	-4.281376
174	H	4.770566	2.057423	-3.333564
175	C	6.875637	-0.340654	-4.505594
176	H	8.996931	-0.413390	-4.115975
177	H	4.757645	0.017592	-4.718635
178	H	6.871908	-1.237490	-5.119871
179	C	6.515540	4.580570	1.610159
180	C	7.602484	4.493977	2.492164
181	C	5.237120	4.773254	2.153622

182	C	7.418315	4.584610	3.872543
183	H	8.603655	4.354878	2.093082
184	C	5.047400	4.863407	3.533246
185	H	4.380708	4.851120	1.489479
186	C	6.138466	4.767767	4.398854
187	H	8.276896	4.515932	4.535690
188	H	4.046636	5.011926	3.930900
189	H	5.992973	4.840839	5.473515
190	C	-6.878419	-1.945874	-2.865783
191	C	-8.060602	-1.228504	-3.098928
192	C	-5.687675	-1.460538	-3.425891
193	C	-8.053406	-0.056676	-3.856794
194	H	-8.996359	-1.592022	-2.682280
195	C	-5.674741	-0.287556	-4.182063
196	H	-4.759834	-2.002830	-3.267514
197	C	-6.858630	0.419861	-4.399188
198	H	-8.983153	0.480870	-4.025664
199	H	-4.738331	0.069942	-4.602316
200	H	-6.850706	1.331318	-4.991443
201	C	-6.533031	-4.622175	1.612512
202	C	-7.629073	-4.563613	2.485586
203	C	-5.257625	-4.811189	2.164398
204	C	-7.456758	-4.677993	3.865775
205	H	-8.628188	-4.427873	2.080095
206	C	-5.079886	-4.924746	3.543901
207	H	-4.394286	-4.867563	1.507129
208	C	-6.179945	-4.856998	4.400740

209	H	-8.322287	-4.630956	4.521693
210	H	-4.081309	-5.069705	3.948404
211	H	-6.043620	-4.948449	5.475203
